# Micro/Nanorobots (MNRs) at the Intersection of Mechanobiology and Artificial Intelligence: An Emerging Frontier for Precision Nanomedicine

**DOI:** 10.1021/acsnanomed.5c00168

**Published:** 2026-06-09

**Authors:** Tahniat Afsari, Jamile Harmouch, Rahul Sharma, Ruisheng Liu, Subhra Mohapatra, Shyam S. Mohapatra

**Affiliations:** † Department of Internal Medicine and Laboratory for Translational Nanotechnology, 33697 Morsani College of Medicine, Tampa, Florida 33612, United States; ‡ Department of Molecular Medicine, 33697 Morsani College of Medicine, Tampa, Florida 33612, United States; § Division of Nephrology, Center for Immunity Inflammation and Regenerative Medicine, University of Virginia School of Medicine, Charlottesville, Virginia 22903 United States; ∥ Department of Molecular Pharmacology and Physiology, 33697 Morsani College of Medicine, Tampa, Florida 33612, United States; ⊥ James A. Haley VA Hospital, Tampa, Florida 33612, United States

**Keywords:** Micronanorobots (MNRs), Microbots, Nanobots, Synthetic MNRs, Biohybrid MNRs, Mechanobiology, Artificial intelligence, Targeted delivery and Glomerular targeting

## Abstract

Micro/nanorobots (MNRs) are emerging as active precision-nanomedicine platforms that convert physical energy into propulsion and localized cellular or tissue mechanotransduction. Yet, the ways in which MNR-mediated forces and flows reprogram cell behavior, activate mechanotransduction pathways, or drive tissue remodeling remain poorly understood. Unlike passive nanocarriers that rely on diffusion and vascular permeability, MNRs enable directional transport, barrier penetration, and spatiotemporal control. Beyond locomotion, propulsion-induced stresses act as mechanical cues that engage cellular mechanosensing pathways, including membrane-tension regulation, ion-channel activation, cytoskeletal remodeling, and downstream intracellular transcriptional signaling. Here, we review MNRs as mechanobiology-driven systems in which propulsion physics is intentionally coupled to force-sensitive biological responses. Rather than categorizing MNRs solely by actuation modality, we propose force-to-function coupling as a unifying design principle linking propulsion dynamics to defined mechanotransduction outcomes at the nano–bio interface. We further discuss how artificial intelligence (AI) introduces predictive modeling, imaging-guided feedback, and closed-loop optimization to achieve programmable biological outputs under physiological uncertainty. This review establishes a framework for biology-aware and intelligent MNRs by integrating propulsion engineering, mechanobiology, and computational control, and highlights key translational challenges that must be addressed to realize precision nanomedicine.

## Graphical abstract

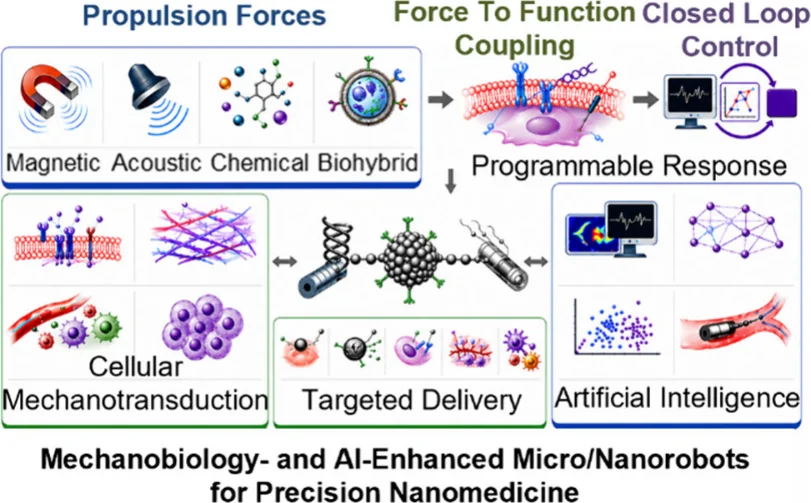

## Introduction

Over the past two decades, nanotechnology has generated diverse range of nanoscale platforms for biomedical delivery, including liposomes, lipid nanoparticles, polymeric micelles, dendrimers, carbon-based nanomaterials, and inorganic systems such as gold, silica, magnetic iron oxides, and quantum dots.^1–5^ These materials enhance drug stability, circulation time, and pharmacokinetic control. Increasingly, design principles have expanded beyond chemical composition to include mechanical and structural parameters, as nanoparticle size, shape, surface chemistry, stiffness, and deformability influence protein corona formation, immune interactions, cellular uptake, and transport through complex biological matrices.^6–8^ At the nanoscale, particle mechanics and mechanical signals regulate biological function and influence biodistribution, phagocytic clearance, circulation half-life, and penetration through dense tumor or fibrotic tissues.^9,10^ Membrane tension, cytoskeletal contractility, and extracellular matrix stiffness further govern cellular adhesion, migration, and cell fate decisions.^7,8^ Thus, mechanobiological interactions at the nano–bio interface can further modulate intracellular signaling, although the causal relationships between applied forces and specific biological responses remain poorly understood.^11–13^ A major limitation of traditional nanosystems is that these mechanobiological interactions are governed by passive transport.^14–16^ Delivery of nanoparticles within the ∼50–150 nm size range^17,18^ relies primarily on diffusion, vascular permeability, and in some contexts, the enhanced permeability and retention effects. Under these conditions, transport occurs in low-Reynolds-number regimes (Re ≪ 1), in which viscous forces dominate and directional control is intrinsically constrained.^15^ As a result, passive systems lack spatiotemporal precision in dynamic physiological environments.

The emergence of micro- and/or nanoscale robots (MNRs) offers a pathway to overcome these constraints because MNRs can convert external or internal energy inputs into sustained propulsion and localized cellular mechanotransduction.^16,17^ Continuous energy input breaks symmetry and overcomes viscous drag, enabling directional locomotion, localized flow fields, and controlled interactions with cellular and tissue barriers.^18–21^ Crucially, propulsion-generated stresses, deformation fields, and shear gradients act as mechanical stimuli that engage mechanosensitive ion channels, integrin–focal adhesion complexes, cytoskeletal networks, and nucleus-associated signaling pathways.^26–29^ Thus, MNR-mediated propulsion both enhances transport and functions as a programmable and sustained source of biological stimulation.^22–25^

In this review, we define MNRs as systems in which propulsion dynamics are deliberately engineered to modulate force-sensitive cellular mechanotransduction processes. We propose force-to-function coupling as a central design principle that links propulsion physics to defined mechanotransduction outcomes. Within this framework, mechanical energy becomes a controllable variable capable of modulating membrane tension, intracellular trafficking, cytoskeletal organization, and transcriptional activity. We further examine how artificial intelligence (AI) adds a computational control layer through predictive modeling, adaptive feedback, and closed-loop optimization of actuation parameters. As MNRs evolve from passive carriers to dynamic mechanobiological interfaces, this review highlights the state-of-the-art in propulsion engineering, mechanobiological interactions, AI-enabled design and control, and translational challenges.

## Engineering and Functionalization of MNRs

MNR performance is determined not only by propulsion modality but also by structural architecture, material composition, and fabrication precision. Engineering decisions at the nanoscale directly influence mechanical output, field responsiveness, biodegradability, and biological compatibility. Accordingly, fabrication strategies must be evaluated through the lens of force generation, structural anisotropy, and translational feasibility rather than geometric control alone. Three major fabrication methods dominate nanomedicine-oriented MNR design: template-assisted electrodeposition, colloidal/wet-chemical synthesis, and hybrid soft–hard architectures tailored for biointegration (Figure 1).

**Figure 1:**
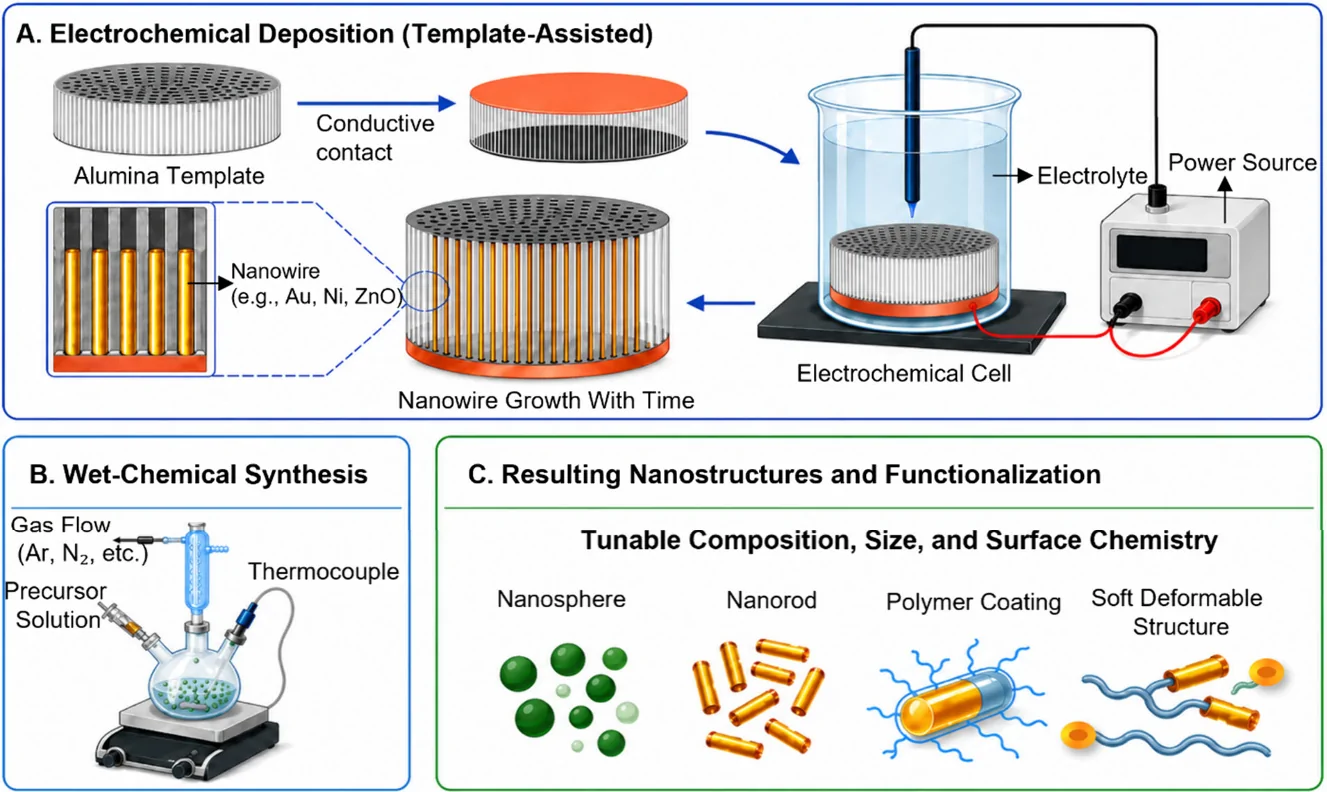
Fabrication routes for tunable nanostructures. (A) Template-assisted electrochemical deposition of nanowires using anodic alumina membranes with conductive backing; nanowire length is controlled by deposition time. (B) Wet-chemical synthesis via controlled precursor injection and temperature regulation. (C) Resulting architectures include nanospheres, nanorods, and polymer-coated or soft deformable structures for engineered nano–bio interactions. Note: this figure is intended solely for conceptual visualization.

### Template-Assisted Electrodeposition

Template-assisted electrodeposition, particularly using anodic aluminum oxide (AAO) membranes, enables fabrication of nanowire with precisely controlled diameter and length (high aspect ratios).^26^ Structural anisotropy is defined by pore geometry, and nanowire length can be tuned by adjusting deposition time. These features allow modulation of torque generation under magnetic actuation and shear production under acoustic fields.^31–33^ However, template-assisted systems often rely on metallic materials such as Au, Ni, Pt, raising concerns regarding long-term persistence, biodegradability, and potential ion release.^27^ Batch-to-batch reproducibility and membrane removal steps can introduce structural variability. For translational deployment, scalable manufacturing and material substitution toward biodegradable or transient systems remain ongoing challenges.^28–30^

### Colloidal and Wet-Chemical Synthesis

Wet-chemical synthesis provides scalable routes to nanospheres, nanorods, Janus particles, and asymmetric structures capable of catalytic or field-responsive propulsion.^29^ Shape anisotropy and surface patterning can be tuned through precursor concentration, ligand chemistry, and reaction kinetics.^35–37^ Despite compositional flexibility, these systems offer limited fine-scale control over mechanical stiffness and elastic modulus.^29^ In biological environments, protein adsorption and surface reconstruction can alter propulsion symmetry and mechanical output.^36–38^ Reproducibility under physiological ionic strength and protein-rich conditions therefore represents a major translational constraint.

### Hybrid and Soft-Matter Architectures

Soft polymeric microrobots, hydrogel-based systems, and membrane-coated constructs introduce deformability and improved biocompatibility.^31^ Elastic mismatch between rigid and soft components can be engineered to modulate membrane wrapping energetics and immune recognition.^9^ Hybrid systems combining metallic cores with polymeric or biomimetic coatings aim to balance propulsion efficiency with biological tolerance.^32,33^ Despite improved safety profiles, soft systems frequently exhibit reduced propulsion efficiency and limited torque transmission. Mechanical fatigue, swelling, and degradation under physiological shear must be considered. Furthermore, fabrication reproducibility for multilayer hybrid systems remains technically demanding.^41–44^

### Structural Anisotropy and Symmetry Breaking

Directed propulsion under low-Reynolds-number regimes requires symmetry breaking.^19,20^ Anisotropic geometries like helical, rod-like, or Janus configurations can convert rotational or field energy into directional translation.^21,45^ Structural asymmetry governs local flow-field generation and determines the spatial distribution of shear and stress at the nano–bio interface. However, geometric anisotropy alone does not guarantee predictable biological responses. Propulsion efficiency depends on coupling between actuation frequency, fluid viscosity, and structural rigidity.^45,46^ Quantitative models linking structural parameters to force amplitude and spatial stress gradients remain underdeveloped, limiting predictive design.^44^

### Surface Functionalization of MNRs for Mechanobiological Performance

Surface functionalization represents the critical interface that converts structurally engineered MNRs into biologically interactive systems. While fabrication strategies such as template-assisted electrodeposition or wet-chemical synthesis define geometry and propulsion capability, surface chemistry dictates colloidal stability, immune evasion, cellular uptake pathways, and mechanotransduction coupling. For metallic MNRs (e.g., Au, Ni, Pt based), thiol–gold chemistry provides a robust platform for modular surface modification. Self-assembled monolayers (SAMs) of thiolated polyethylene glycol (PEG) reduce opsonization and prolong circulation by minimizing protein corona formation.^34,35^ PEG chain length and grafting density regulate hydrodynamic radius and steric stabilization, directly influencing vascular margination and tissue penetration.^36^ In mechanobiology-driven systems, PEGylation must be optimized to balance stealth performance with sufficient membrane interaction to permit force transmission under acoustic or magnetic actuation.

Polymeric and lipid-based coatings extend functionality beyond passive stabilization. Lipid bilayer coatings, inspired by lipid nanoparticles (LNPs), enable electrostatic or ionizable encapsulation of nucleic acids while preserving actuation capability.^6^ However, lipid composition must be tuned to avoid complement activation and infusion-related reactions, particularly in systemic administration. Biodegradable polymers such as PLGA, poly­(β-amino ester) (PBAE), chitosan, or polyamidoamine (PAMAM) dendrimers allow cargo complexation and pH-responsive release.^37,38^ Cationic surfaces can enhance endosomal escape through proton sponge effects but may elevate cytotoxicity and inflammatory signaling, necessitating careful surface-charge modulation.

Biomimetic functionalization strategies further improve in vivo performance. Cloaking MNRs with red blood cell (RBC) or platelet-derived membranes imparts CD47-mediated “self” recognition, reducing macrophage uptake and prolonging circulation.^39^ Platelet membrane coatings additionally confer affinity for inflamed endothelium or tumor-associated vasculature, aligning targeting specificity in disease microenvironments.^40–42^ Such bioinspired interfaces are particularly relevant for mechanotransduction-driven MNRs, where sustained and intimate membrane contact is required for effective force-mediated signaling.

Collectively, surface functionalization strategies convert rigid propulsion units into dynamic biointerfaces capable of immune modulation, targeted adhesion, controlled release, and mechanobiological engagement. In mechanobiology-driven MNRs, surface engineering is not merely a biocompatibility step, it is a functional design axis that determines how external physical forces are translated into cellular responses and therapeutic outcomes.

## Mechanobiology Centric Design Principles Governing Force–Cell Interactions of MNRs

MNRs induce mechanobiological effects, i.e., mechanotransduction, a process by which cells detect mechanical forces and convert them into biochemical signals that regulate cellular function, gene expression, and fate decisions. Specifically, MNRs exert mechanical forces through cues such as shear stress, tensile strain, matrix stiffness, hydrodynamic drag, and acoustic radiation forces applied at the membrane or cytoskeleton, which regulate cellular function through defined mechanosensing pathways capable of transducing physical inputs into biochemical signaling.^11–13^ Unlike concentration-dependent biochemical signaling, mechanotransduction depends on the magnitude, direction, duration, and spatial alignment of applied forces relative to force-sensitive structures. Central to mechanotransduction are integrin-based adhesion complexes, which physically link the ECM to the actin cytoskeleton and act as molecular clutches that transmit and regulate forces across the cell boundary at nanometer- to micrometer-scale domains.^60–63^ Such mechanical loading alters protein conformation within these complexes such as mechanosensitive ion channels (e.g., Piezo1, TRPV4), actomyosin tension networks, and nucleus–lamina coupling, which leads to triggering downstream signaling pathways such as focal adhesion kinase (FAK), Rho GTPases, and transcriptional regulators including YAP/TAZ, thereby coupling mechanical input to long-term functional outcomes (Figure 2).

**Figure 2:**
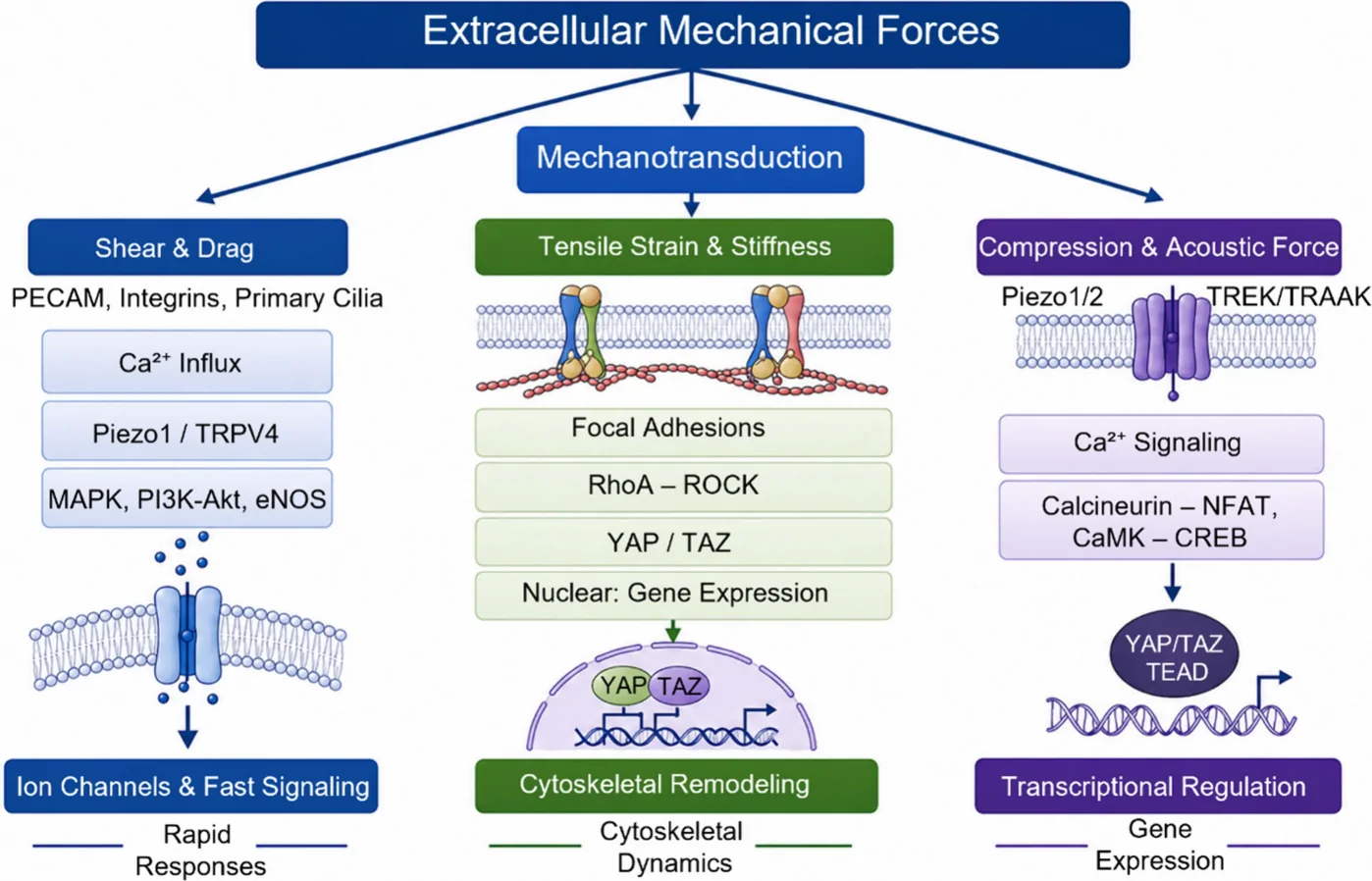
Systematic linkage between physical forces and mechanotransduction pathways. Mechanical cues including shear stress, tensile strain, matrix stiffness, hydrodynamic drag, and acoustic radiation forces, activate distinct classes of cellular mechanosensors. Shear and drag primarily stimulate apical mechanosensors (PECAM-1, integrins, primary cilia) and mechanosensitive ion channels (Piezo1, TRPV4), inducing rapid Ca^2+^ influx and downstream MAPK, PI3K–Akt, and eNOS signaling. Tensile strain and stiffness regulate focal adhesions and cytoskeletal tension via RhoA–ROCK, promoting YAP/TAZ nuclear localization and cytoskeletal remodeling. Compression and acoustic forces activate Piezo1/2 and TREK/TRAAK channels, triggering Ca^2+^-dependent pathways (calcineurin–NFAT, CaMK–CREB) that converge on YAP/TAZ-TEAD-mediated gene expression. Together, these mechanotransduction axes provide a unified framework linking biophysical stimuli to cellular decisions. Note: this figure is intended solely for conceptual visualization.

### Force Requirements and Cellular Sensitivity to Mechanical Inputs

Specifically, for MNR systems, propulsion-generated stresses function as externally programmable mechanical inputs that interface with intracellular mechanotransduction pathways. Cells are highly sensitive to forces at the pico- to nanonewton scale, with individual receptor–ligand bonds responding to forces as low as 1–10 pN.^34^ Integrins and associated adaptor proteins such as talin and vinculin undergo force-dependent unfolding at similar magnitudes, enabling mechanotransduction to occur without gross cell deformation.^35^ Mechanosensitive ion channels, particularly PIEZO1 and PIEZO2, gate in response to membrane tension and curvature, converting mechanical perturbations into rapid calcium influx and electrical signaling. Experimental studies using molecular tension sensors and magnetic nanoparticle actuation demonstrate that sustained or oscillatory forces within this physiological range are sufficient to activate signaling cascades, whereas excessive loading can disrupt membrane integrity or induce cytotoxicity.^34,35^

### Mechanical Interactions as a Design Driver for MNRs Because

mechanotransduction depends strongly on force magnitude, directionality, and dynamics, mechanical interactions play a defining role in the design of magnetically actuated nanorobots (MNRs). Thus, rational design therefore requires quantitative calibration of force amplitudes, spatial distribution, and temporal patterning to achieve biological activation without inducing cytotoxic deformation.^57–60^ Effective MNR platforms must generate forces that match the activation thresholds of specific mechanosensors while maintaining spatial precision and biocompatibility. Particle size, geometry, and magnetic anisotropy determine whether applied fields produce translational forces, torque, or localized membrane deformation. For example, disk- or rod-shaped magnetic particles convert alternating magnetic fields into rotational or oscillatory stresses that more efficiently engage mechanosensitive receptors than spherical particles. Consequently, MNRs are increasingly engineered to apply dynamic, receptor-targeted mechanical stimuli rather than relying on bulk forces or static loading.^36,37^ Critical determinants of MNR-induced mechanotransduction such as, force magnitude and activation thresholds, directionality and specificity, mechanical memory, spatial alignments, mechanical vulnerability and selective mechanotherapy are described below.

### Force Magnitude and Activation Thresholds

Cells operate within well-defined mechanical windows. Piconewton-scale forces activate integrin clusters and mechanosensitive ion channels, whereas cytoskeletal contractility typically occurs in the nanonewton range.^38–40^ Subthresholds forces fail to initiate signaling, whereas excessive forces can rupture the cytoskeleton or compromise membrane integrity.^41^ Nuclear deformation provides a quantitative benchmark: ∼5–10% nuclear strain alters chromatin organization and transcription, while larger deformations increase the probability of nuclear envelope rupture and DNA damage.^42^ Ultrasound-driven microrobots generate shear stresses of ∼0.1–10 Pa depending on acoustic frequency and pressure, a range known to activate endothelial integrin signaling and nitric oxide production.^43,44^ However, force magnitude alone does not determine biological response; directional and temporal encoding critically modulate outcomes.^51^

### Directionality and Mechanosensory Specificity

Mechanotransduction is inherently receptor- and context-dependent, reinforcing the importance of surface functionalization and actuation mode in MNR design. Functionalization with ligands for integrins, ion channels, or other mechanoreceptors enables externally applied magnetic fields to deliver localized forces directly to signaling hubs. Studies using integrin- or PIEZO-targeted magnetic nanoparticles show that oscillatory forces of only a few piconewtons per particle can elicit robust downstream responses, including calcium signaling, differentiation, and tissue remodeling. Importantly, dynamic stimulation—rather than static force application—produces stronger and more sustained biological effects, highlighting the need for temporal control in MNR actuation strategies.^45,46^ Also, mechanical cues are inherently directional. Tangential shear stress preferentially activates endothelial mechanosensitive ion channels and nitric oxide signaling.^43^ Compressive stress alters nuclear morphology and chromatin organization.^47^ Tensile strain reorganizes focal adhesions and actin stress fibers through integrin-mediated tension transmission.^48^ While passive nanoparticles primarily exert isotropic adhesive forces, MNRs impose directional shear fields, rotational torque, or oscillatory strain gradients.^49^ Such vectorial actuation enables selective engagement of curvature-sensitive membrane domains, integrin clusters, and cytoskeletal filaments, yielding mechanobiological specificity unattainable with static systems.^50^

### Temporal Encoding and Mechanical Memory

Mechanotransduction is temporally encoded. Mechanosensitive ion channels respond within milliseconds to oscillatory forces.^51^ Sustained tension over minutes to hours promotes cytoskeletal reinforcement and transcriptional reprogramming via YAP/TAZ and associated pathways.^42^ Passive nanocarriers lack such temporal programmability. In contrast, acoustically and magnetically actuated MNRs permit frequency-tuned stimulation capable of selectively engaging rapid ion-channel responses or slower transcriptional pathways.^21^ Frequency encoding thus introduces a powerful design parameter for modulating mechanically resistant disease phenotypes.

### Length-Scale Matching and Spatial Coupling

Effective mechanotransduction requires spatial alignment between applied forces and mechanosensitive structures. Membrane curvature sensing occurs at tens-of-nanometer scales, focal adhesions span submicrometer domains, cytoskeletal networks operate at micrometer scales, and nuclear deformation involves whole-nucleus strain fields.^60–62^ Bulk mechanical therapies lack cellular precision, whereas passive nanoparticles often generate insufficient deformation to engage mechanosensitive architecture.^9^ MNRs bridge this scale gap by generating localized stress gradients comparable to cellular dimensions.^20,21^ However, quantitative mapping of propulsion parameters to intracellular stress distribution remains incomplete.

### Mechanical Vulnerability and Selective Mechanotherapy

Healthy cells maintain mechanical homeostasis through active modulation of cytoskeletal tension and membrane mechanics.^40,42^ Diseased cells, however, exhibit altered stiffness, abnormal contractility, and compromised nuclear integrity, lowering their mechanical failure thresholds.^41^ MNRs can exploit this vulnerability by delivering forces tolerated by healthy cells but exceeding the adaptive capacity of diseased cells.^42,43,52^ This principle provides a mechanobiological basis for selective mechanotherapy, independent of receptor-based targeting. Further, careful calibration of force amplitude and exposure duration remains essential to avoid off-target tissue damage. Examples of force-sensor coupling in modulating mechanical microenvironments are shown in Table 1.

**Table 1:** Examples of Force–Sensor Coupling in Modulating Mechanical Microenvironments

Cellular Context	Mechanical Cue	Primary Mechanosensor	Dominant Biophysical Effect	Therapeutic Implication
Tumor microenvironment^40^	ECM stiffening, shear, confinement	Integrin–FAK complexes	Elevated traction forces; reduced stress relaxation	Matrix normalization; improved transport
Cancer cell cortex/nucleus^11,53^	Membrane tension; compression	Actin cortex; lamin A/C	Nuclear deformation; cytoskeletal failure	Selective mechanoptosis
Stromal cells^10,12^	Cyclic strain; matrix tension	Integrins; focal adhesions	Myofibroblast activation	Tumor microenvironment remodeling
Immune cells^13^	Curvature stress; confinement	Piezo1; TRPV4	Ca^2+^ influx; cortical remodeling	Immune reprogramming
Nano–bio interface^42,50^	Elasticity mismatch	Curvature-sensitive endocytic machinery	Altered membrane wrapping energetics	Elasticity-encoded uptake
Macrophages (RES)^54^	Large deformation	Cytoskeleton; clathrin	Transient uptake suppression	Prolonged circulation

Together, advances in mechanotransduction research indicate that mechanical force itself can function as a primary biological signal, not merely a secondary physical effect. This understanding establishes clear design constraints for MNR systems: forces must be within physiological ranges, delivered dynamically, and coupled to relevant mechanosensors in the target tissue. By explicitly integrating mechanobiological principles into MNR engineering, it becomes possible to modulate cellular behavior with high specificity while minimizing off-target biochemical effects. As a result, mechanotransduction-informed MNR design offers a powerful framework for therapeutic modulation, tissue engineering, and mechanistic interrogation of cellular force sensing.^34,55^

## Propulsion Modes as Distinct Mechanical Signatures

Propulsion modality determines not only how an MNRs move but also the spatial distribution, amplitude, and temporal characteristics of mechanical stress imparted at the nano–bio interface. Each actuation strategy generates a distinct force field such as shear, torque, compressive oscillation, or reaction-driven gradients that differentially engage mechanosensitive pathways. Accordingly, propulsion should be interpreted as a mechanical signature rather than merely an energy input. Propulsion mechanisms of MNRs driven by chemical, magnetic, and acoustic actuation are illustrated in Figure 3. Also, a comparative overview of propulsion modes and associated mechanobiological effects are summarized in Table 2. In addition, the nanomedicine application of these various MNR propulsion modes, with the corresponding energy source and dominant signatures at the interface is summarized in Table S1.

**Figure 3:**
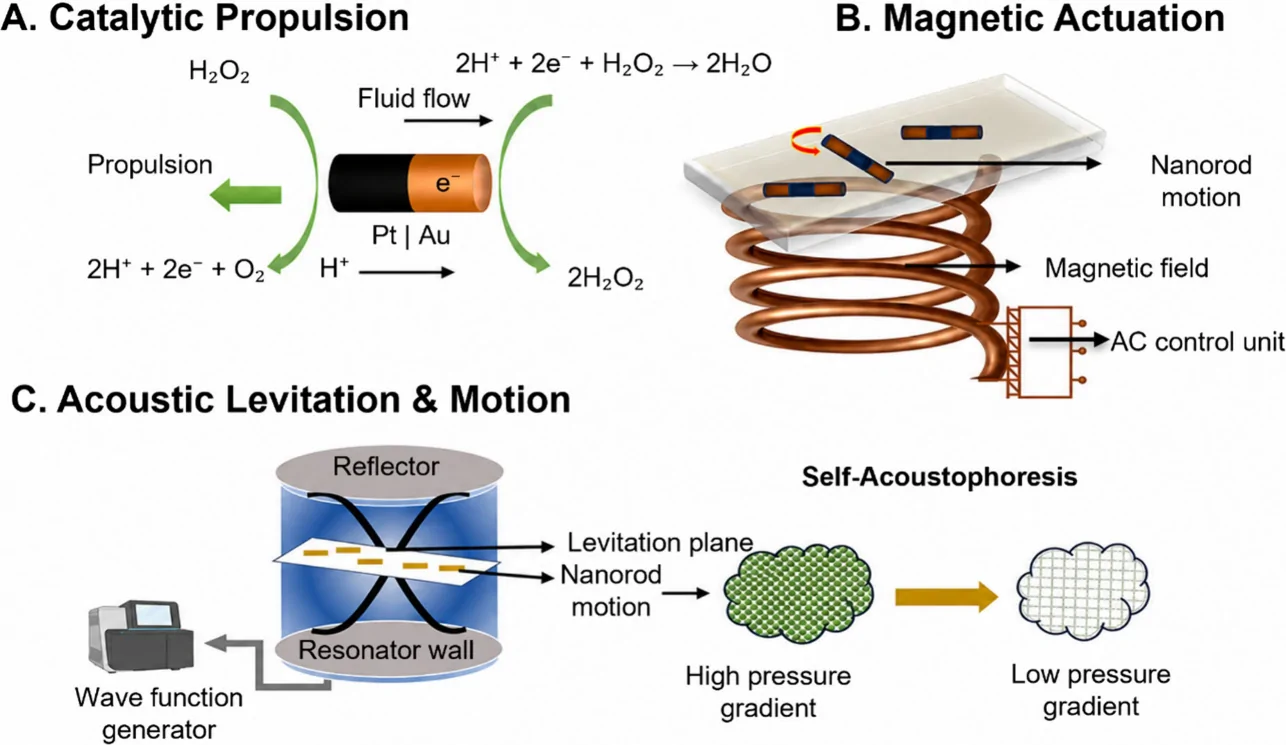
Propulsion mechanisms of MNRs driven by chemical, magnetic, and acoustic actuation. (A) Catalytic self-propulsion of a bimetallic nanorod in hydrogen peroxide, where asymmetric redox reactions at Pt and Au segments generate ionic gradients and fluid flow that drive motion. (B) Magnetic-field-driven actuation, in which an external alternating magnetic field induces rotational and translational motion of magnetic nanorods. (C) Acoustic propulsion via self-acoustophoresis inside a resonant chamber, where standing pressure waves create pressure gradients that move particles along the levitation plane from high- to low-pressure regions. Note: this figure is intended solely for conceptual visualization.

**Table 2:** Comparison of Propulsion Modes and Mechanobiological Effects

Propulsion Mode	Energy Source	Dominant Force Signature	Primary Mechanobiological Interaction	Translational Strength	Key Limitation
Ultrasound^56–58^	Acoustic waves	Oscillatory shear, radiation force	Transient membrane deformation; junction modulation	Deep tissue penetration; BBB opening	Cavitation safety thresholds
Magnetic^59–62^	External magnetic fields	Torque, directional shear	Controlled positioning; localized mechanical interaction	MRI compatibility; precise steering	Requires external hardware
Catalytic^63–66^	Chemical fuels	Diffusiophoretic thrust; bubble recoil	Reaction-driven shear; local deformation	Autonomous motion; GI delivery	Limited directional control
Optical^67–70^	NIR/visible light	Thermal gradients; surface charge asymmetry	Local membrane perturbation; ROS generation	Spatiotemporal precision	Limited depth penetration

### Acoustic Propulsion: Oscillatory Shear and Microstreaming

Acoustic and ultrasound-driven MNRs exploit acoustic radiation forces, bubble oscillation, and microstreaming to generate propulsion.^21^ As acoustic waves propagate through fluid, they produce oscillatory pressure gradients, radiation forces, and microstreaming vortices.

In asymmetric MNRs such as Janus particles, microbubbles, or multisegmented nanorods, these oscillatory forces are rectified into net propulsion through drag differentials and boundary streaming. Reported velocities reach several hundred μm·s^–1^ under MHz-range excitation.^14,49,71^

Acoustic actuator modeling, system identification, trajectory control, and closed-loop manipulation are central to treating acoustically driven micro- and microrobotic systems as controllable robotic platforms. Acoustic field modeling typically starts from linear acoustics, solving the Helmholtz equation to predict pressure and velocity fields generated by piezoelectric transducers, with radiation forces and acoustic streaming derived to describe forces and torques on particles or microrobots.^72^ In practice, deviations caused by transducer coupling, boundary reflections, temperature dependence, and fluid loading motivate system identification, where experimentally measured particle responses or pressure fields are used to calibrate effective force models.^73^ These models enable trajectory control through spatiotemporal modulation of acoustic phase, frequency, or amplitude to guide robots along prescribed paths. Closed-loop manipulation further improves accuracy by incorporating real-time visual or sensor feedback to correct for drift, flow disturbances, and modeling error, enabling robust positioning and repeatable motion.^74^ Key challenges in closed-loop acoustic control include limited sensing bandwidth at microscale, strong coupling between multiple robots, nonlinear streaming effects, and the computational complexity of updating full acoustic field models in real time, which remain active areas of research.

Beyond propulsion, ultrasound generates transient mechanical stresses capable of modulating endothelial junctions and membrane tension. Focused ultrasound (FUS), when paired with microbubbles, induces reversible opening of the blood–brain barrier (BBB) via cavitation-mediated tight-junction remodeling.^57,75^ These effects directly engage mechanotransduction pathways associated with membrane deformation, calcium influx, and cytoskeletal signaling.

Lipid-shelled microbubble MNRs can self-assemble and migrate upstream against physiological flow, demonstrating propulsion under high-shear vascular conditions.^76,77^ Ultrasound propulsion therefore couples directional transport with transient membrane permeabilization and vascular modulation. However, acoustic fields attenuate with tissue depth and may induce off-target heating at higher intensities. Precise calibration of acoustic pressure is therefore essential to maintain mechanobiological specificity.

### Magnetic Actuation: Torque-Driven Translation and Rotational Stress

Magnetically actuated MNRs convert external magnetic fields into translational or rotational motion through magnetic dipole alignment (τ = m × B).^72–74^ Under low-Reynolds-number conditions, rotation of helical or anisotropic structures generates propulsion through viscous coupling.^20,21^ Magnetic actuation enables remote, field-directed manipulation without direct energy deposition into tissue. Magnetic torque and field gradients act on dipole moments embedded in Fe_3_O_4_-, Ni-, or Co-containing MNRs,^78^ enabling rotating, gradient-driven, or oscillatory propulsion modes depending on the applied fieldmodes.^49^

Magnetic actuation modeling, system identification, trajectory control, and closed-loop manipulation are foundational to robotic magnetic nano- and microrobot (MNR) operation. Actuation modeling links applied magnetic fields to forces and torques on the robot by combining electromagnetic field models with mechanical and hydrodynamic dynamics. In engineered magnetic micromanipulator systems, this modeling explicitly incorporates actuator geometry, field gradients, nonlinearities, and cross-coupling, enabling predictive control rather than qualitative steering. System identification further refines these models by experimentally calibrating uncertainties such as coil misalignment, current–field mappings, and viscous effects, producing usable actuator-level models for control design.^79^

Building on accurate models, trajectory control and closed-loop manipulation allow magnetic robots to follow time-varying paths with feedback compensation for disturbances and modeling errors. Vision- or sensor-based feedback enables real-time correction using classical or model-based control strategies, transforming magnetic MNRs into controllable robotic systems. Studies such as Ablay et al., demonstrate this end-to-end approach, integrating full actuator modeling, identification, and feedback control within engineered magnetic micromanipulators, moving beyond purely physical descriptions of magnetic actuation toward reproducible, scalable robotic manipulation.^80^ Examples of different magnetic actuator systems are provided below.

Rotating magnetic fields drive helical microrobots in corkscrew-like trajectories, enabling precise navigation within confined microvascular environments. Field gradients provide translational guidance, while oscillatory fields induce surface rolling or tumbling movements along surfaces. Unlike acoustic propulsion, magnetic actuation primarily generates torque and shear at cell-robot interface rather than bulk cavitation.^81,82^ Also, hydrogel-based capsule microrobots controlled by multiaxis magnetic platforms can transport therapeutic cargo and release payloads under tunable magnetic conditions.^83^ Thermoresponsive magnetic systems enable triggered release at defined temperature thresholds.^59^ Further, magnetic propulsion provides high spatial precision, compatibility with MRI-based navigation, and deep-tissue penetration. The shear stresses generated by rotating microrobots can reach physiologically relevant levels depending on field strength and structural geometry. These features make magnetic actuation particularly attractive for targeted mechanotherapeutic applications.

### Catalytic Propulsion: Reaction-Driven Gradients

Chemical and catalytic actuation is typically modeled by coupling reaction kinetics at the robot surface (e.g., fuel decomposition on catalytic coatings) with diffusion and advection of chemical species in the surrounding fluid, yielding concentration fields that generate propulsion through self-diffusiophoresis or self-electrophoresis.^84^ Catalytic MNRs convert chemical gradients into directed motion through asymmetric surface reactions. Interfacial concentration gradients generate self-diffusiophoretic propulsion, whereas gas evolution produces bubble recoil forces that propel the robot forward.^21^ Although, hydrogen peroxide decomposition remains the most widely used model reaction, endogenous fuels such as glucose or urea are preferred for biomedical applications because they avoid exogenous chemical introduction.^71^ Metal-based systems, including magnesium (Mg) and zinc (Zn) micromotors, exploit acidic gastrointestinal conditions to generate hydrogen bubbles and achieve autonomous propulsion for localized GI delivery.^85^ Catalytic propulsion produces localized chemical gradient formation and mechanical thrust without external field. However, directional control is limited, and propulsion efficiency depends heavily on local fuel availability, which reduces reproducibility in complex biological environments.^86^ From mechanobiological perspective, catalytic systems primarily generate localized shear stresses and reaction-driven deformation at the nanobio interface.^63^

Examples of chemical and catalytic actuator modeling, system identification, trajectory control, and closed-loop manipulation illustrate how chemically powered micro- and microrobots can be treated as controlled robotic systems despite their inherently nonlinear and stochastic actuation. In practice, system identification is used to estimate effective propulsion coefficients, fuel consumption rates, and drag by observing robot velocities under controlled gradients or fuel concentrations, accounting for variability in surface chemistry and environmental conditions.^86^ Trajectory control is commonly achieved by modulating external chemical gradients, fuel availability, or combining chemical propulsion with auxiliary fields, while closed-loop manipulation uses visual feedback to compensate for drift, Brownian motion, and disturbances, significantly improving positioning accuracy and repeatability.^87^ Despite promising in vitro and localized in vivo demonstration, most catalytic propulsion platforms remain unsuitable for clinical use, and demonstrate limited control over propulsion onset and duration. These constraints represent key translation barriers that must be addressed for broader clinical applicability. Key challenges for closed-loop control include slow and spatially diffuse chemical field dynamics, difficulty in measuring concentration fields in real time, strong sensitivity to environmental fluctuations, and intrinsic coupling between propulsion and steering, which make high-bandwidth, multirobot control an ongoing research challenge in chemical microrobotics.

### Light-Driven Photothermal Actuation

Light-responsive MNRs convert optical energy into propulsion via photothermal gradients or photochemical reactions. Optical actuation is commonly modeled using electromagnetic field theory, where laser beam profiles are described using Gaussian optics or diffraction theory and forces are derived from momentum transfer, including gradient and scattering forces as formulated in ray-optics or Maxwell stress tensor frameworks.^66^ In practice, system identification is used to calibrate trap stiffness, force constants, and viscous drag by observing particle responses to known perturbations, accounting for aberrations, misalignment, and medium-dependent effects.^88^ These identified models enable trajectory control through time-varying beam steering, holographic optical tweezers, or intensity modulation to guide particles along desired paths.

Examples of optical actuators include localized heating can induce thermophoretic motion and generate transient membrane perturbation.^89^ Near-infrared (NIR) wavelengths (700–900 nm) provide improved tissue penetration of MNRs while minimizing photodamage.^90^ Gold nanorods can generate self-thermophoretic motion under NIR irradiation, while simultaneously providing photothermal therapy.^91^ Janus architectures-based TiO_2_- or BiVO_4_-create surface-charge asymmetry and reactive oxygen species upon illumination.^92^ Photoisomerization systems using azobenzene create reversible-interfacial tension gradients, providing controllable propulsion.^67^

Optical propulsion of MNRs offers rapid on–off control and spatial precision. Mechanobiologically, these systems couple localized thermal stress and membrane perturbation to produce directed motion. Closed-loop manipulation further improves accuracy by incorporating real-time visual feedback to correct for Brownian motion, drift, and modeling error, enabling stable positioning and high-precision tracking. Key challenges in closed-loop optical control include sensing and control bandwidth limits, stochastic thermal forces at small scales, optical crosstalk in multirobot systems, and computational delays when updating complex field or hologram models in real time, which remain active areas of research in optical microrobotics.

### Biohybrid MNRs Systems

Biohybrid MNRs integrate synthetic architectures with living cells or biological membranes derived from erythrocytes, microalgae, leukocytes, bacteria, sperm and DNA-based structures. These systems combine programmable actuation with native biological functionalities such as endogenous motility, chemotaxis, membrane fusion, and immune evasion mechanisms, enabling improved navigation and tissue specificity.^93^ The key characteristics of these biohybrid platforms are summarized in Table 3. Unlike fully synthetic systems, biohybrid MNRs leverage intrinsic cellular mechanosensing and cytoskeletal machinery to generate propulsion or respond to external fields. External actuation like magnetic, acoustic, or catalytic can further amplify intrinsic motility and modulate force transmission at the cell–tissue interface.^103–105^

**Table 3:** Characteristic Features of Biohybrid MNR Platforms

Biological Component	Propulsion Mode	Mechanobiological Mechanism	Translational Advantage	Primary Challenge
Magnetotactic bacteria^94–96^	Self + magnetic	Flagellar thrust + field alignment	Autonomous navigation	Biosafety control
Macrophage membranes^97,98^	Magnetic/field-assisted	Immune mimicry; reduced clearance	Prolonged circulation	Manufacturing consistency
Sperm cells^99,100^	Flagellar propulsion	Flow-responsive thrust; membrane fusion	High propulsion efficiency	Limited targeting specificity
Microalgae^101,102^	Flagellar swimming	Sustained low-shear propulsion	Pulmonary distribution	Environmental sensitivity
Enzyme-coated MNRs^63^	Catalytic	Substrate-gradient propulsion	Endogenous fuel usage	Limited directional control

Biohybrid MNRs offer three distinct advantages: immune camouflage through membrane mimicry, intrinsic motility via flagella or cytoskeletal propulsion, and enhanced barrier penetration through active mechanical navigation.^93,104,105^ Despite these advantages, translational challenges remain, including biosafety management, manufacturing reproducibility, immune compatibility, and long-term stability.

Biohybrid nano- and micro-robotic systems, which integrate living organisms such as bacteria, sperm cells, or muscle tissues as actuators, rely on actuator modeling, system identification, trajectory control, and closed-loop manipulation to achieve controllable robotic behavior. Biohybrid field modeling typically combines biological activity models, such as flagellar propulsion, chemotactic sensing, or muscle contraction dynamics, with physical frameworks including low-Reynolds-number hydrodynamics and biochemical transport equations to predict motion in response to chemical, optical, or electrical stimuli.^93,106^ Because biological actuation exhibits variability, adaptation, and fatigue, system identification is often performed experimentally to estimate effective propulsion forces, response gains, and stochastic noise characteristics from observed trajectories. Trajectory control is achieved by shaping external cues such as chemical gradients, light patterns, or electric fields, while closed-loop manipulation employs real-time visual feedback to correct deviations due to biological variability, environmental disturbances, and modeling error, significantly improving accuracy and repeatability. However, closed-loop control challenges remain substantial, including delayed and nonlinear biological responses, limited observability of internal states, strong stochasticity at the microscale, and the difficulty of achieving high-bandwidth control without affecting cell viability, making robust and scalable control an ongoing research challenge in biohybrid microrobotics.

Synthetic microrobots typically rely on chemical fuels or magnetic field-based propulsion, enabling millimeter-to-centimeter scale penetration depth and supporting applications such as cancer targeting and thrombosis dissolution. However, these platforms are constrained by fuel depletion, limited biodegrability, and potential material-related toxicity. Biohybrid microrobots, in contrast, leverage ATP-driven cellular energy and endogenous biochemical gradients, which facilitate deeper tissue penetration and make them well-suited for infection control, and regenerative therapies. Despite these benefits, biohybrid systems face challenges arising from biological complexity, batch-to-batch variability, and immune recognition. This comparative framework shown in Figure 4 highlights how propulsion mode, control strategy, and degree of biological integration influence therapeutic suitability and shape translational barriers across disease contexts.

**Figure 4:**
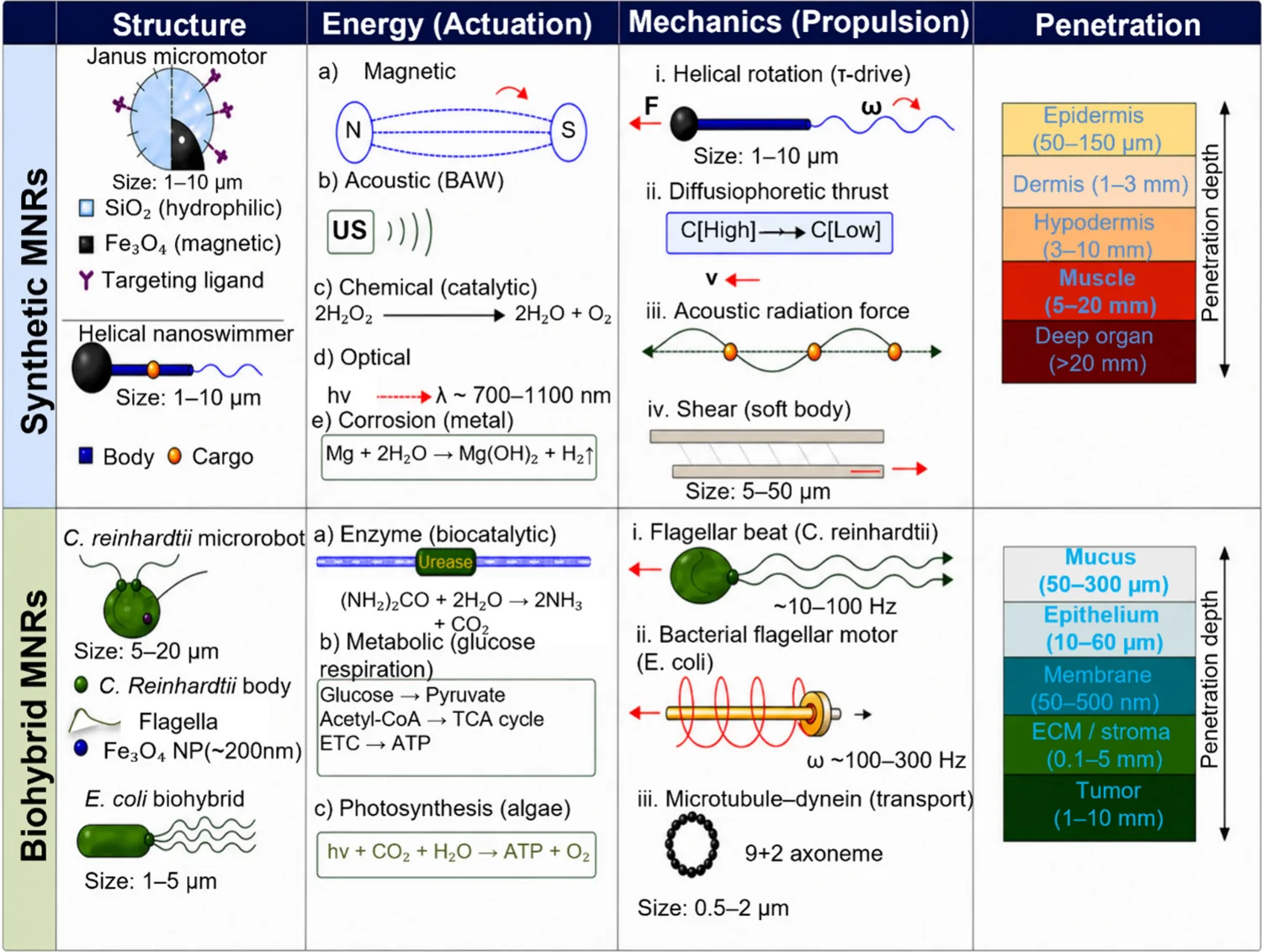
Design and functional characteristics of synthetic and biohybrid MNRs. A comparison of the synthetic and biohybrid MNRs in terms of fuel sources, control modalities, penetration depth, representative disease indications, and associated limitations. Note: this figure is intended solely for conceptual visualization.

An illustrative example of biohybrid MNR-mediated glomerulus-specific drug delivery involves the use of cell-driven or membrane-coated microrobots engineered to navigate renal microvasculature and selectively accumulate within inflamed glomeruli. In this approach, a biohybrid microrobot, such as one powered by a picoeukaryotic cell or coated with endothelial-mimicking membranes, are functionalized with glomerular-targeting peptides or ligands that recognize podocyte or mesangial cell markers exposed during immune-mediated injury (Figure 5).^107^ Actuated by external magnetic or acoustic fields, the MNRs actively traverses the glomerular capillary network, resists rapid washout under high intraglomerular blood flow, and anchors locally through ligand–receptor interactions. Once retained, disease-responsive mechanisms (e.g., inflammation- or ROS-triggered release) enable localized delivery of immunosuppressive or anti-inflammatory drugs directly within the glomerulus. This strategy achieves high local efficacy while minimizing systemic exposure.^107,108^ This biohybrid platform exemplifies how active propulsion, biomimicry, and molecular targeting can be integrated to overcome the limitations of conventional therapies in treating glomerular disease. MNRs achieve glomerular targeting through a combination of size-restricted (typically <150 nm) localization, ligand and peptide functionalization, active navigation and retention and disease specific drug release capabilities that are not possible with standard traditional nanotherapeutic approaches.

**Figure 5:**
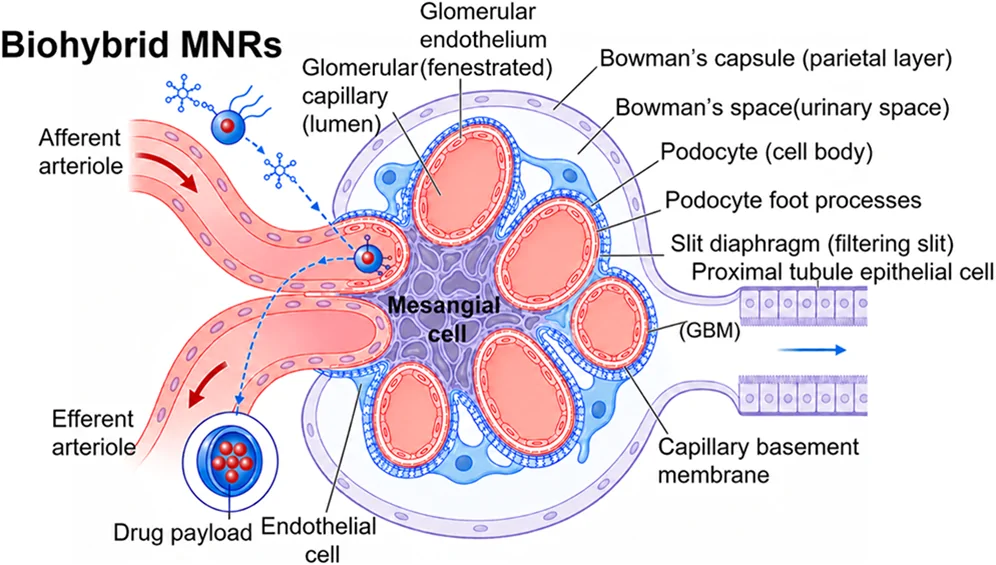
Targeted delivery to the kidney glomerulus using biohybrid MNRs. Schematic illustration of ultrasmall biohybrid microrobots actively navigating the renal vasculature and interstitial spaces toward the glomerulus. The microrobots are propelled by flagella, enabling movement through narrow capillaries under physiological flow conditions. Their surfaces are functionalized with glomerulus-targeting peptides, depicted as molecular ligands that selectively bind to receptors on glomerular endothelial cells and podocytes. Drug-loaded nanoparticles attached to the microrobots are shown with subtle fluorescence to indicate controlled and localized therapeutic delivery. A cross-section of the glomerulus highlights key anatomical features, including capillary loops, the glomerular basement membrane, podocytes, and filtration slits. Note: this figure is intended solely for conceptual visualization.

By combining active renal navigation, molecular targeting, prolonged retention, and controlled drug release, MNRs represent a mechanistically superior therapeutic strategy for treating immune-mediated glomerular diseases, diabetic nephropathy, and other conditions where sustained and localized glomerular treatment is essential but remains challenging with conventional pharmacologic therapies.

## Biomedical Applications and Clinical Relevance of MNRs

MNRs offer unprecedented capabilities for precision intervention, targeted therapy, and real-time sensing within the human body. Their ultrasmall size enables navigation through complex biological environments, enabling access to deep tissues, direct delivery of therapeutics to diseased sites, microscale surgical manipulation, and highly specific interactions with cells and biomolecules.^109,110^ Emerging experimental and preclinical evidence highlights the transformative potential of MNR technologies across major clinical domains including oncology, neurology, gastroenterology, cardiovascular medicine, and regenerative therapy, where they can enhance treatment specificity, reduce systemic toxicity, and support minimally invasive procedures not feasible with conventional clinical tools. Figure 6 summarizes representative MNR modalities, leading clinical or intended applications, and anticipated clinical benefits in key biomedical fields.

**Figure 6:**
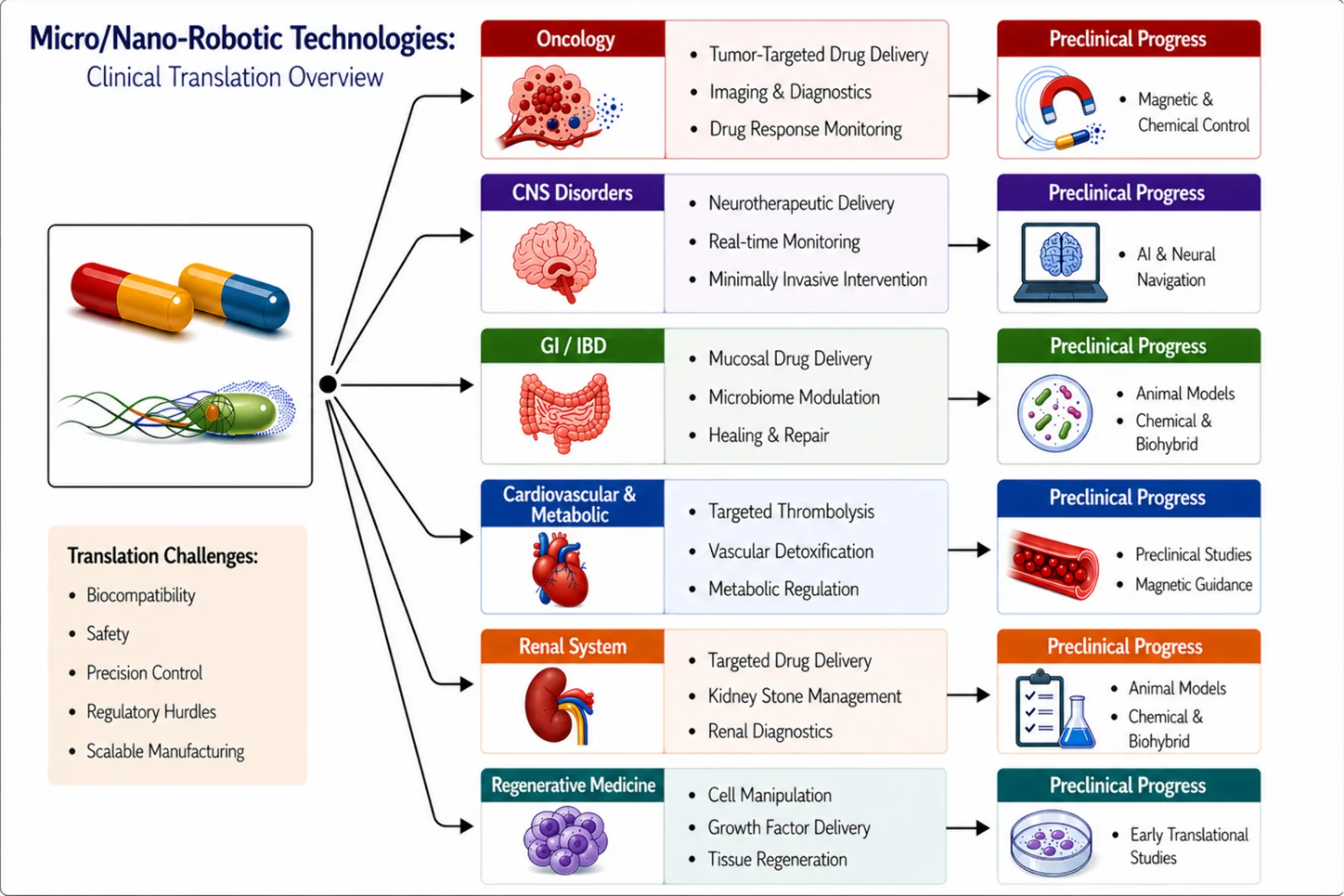
Micro/Nanorobotic technologies: Clinical translation overview. The schematic summarizes an overview of emerging biomedical applications of micro- and nanorobotic systems across five major clinical domains. Each panel highlights representative robotic platforms, key therapeutic and diagnostic applications, current translational readiness, and the dominant propulsion or navigation strategies used. The panels are Oncology, CNS Disorders, GI/IBD, Cardiovascular and Metabolic, Kidney disease, and Regenerative Medicine. Note: this figure is intended solely for conceptual visualization.

To date, in oncology area, preclinical studies demonstrate enhanced tumor localization, improved intratumoral distribution, and controlled payload release using magnetically, acoustic, and chemically propelled microbots. For CNS Disorders MNR Platforms at the concept-stage, with growing research focused on autonomous neural navigation, targeted neuromodulation, and minimally invasive cerebrospinal delivery. In the gastrointestinal diseases including inflammatory bowel diseases, animal-model evidence supports targeted therapeutic delivery and mucosal interaction within complex GI environments, enabled by magnetic and chemical propulsion. In the cardiovascular and metabolic disease areas, large-animal studies indicate the feasibility of organ-scale navigation for clot dissolution, vascular targeting, and metabolic modulation. The regenerative medicine sector is the most advanced in which early translational progress includes microbots-assisted cell delivery, tissue scaffolding and localized biochemical stimulation. Taken together, the advances made in applying MNR technologies to different diseases areas illustrate the broad clinical potential of micro/nanorobotic technologies and highlight how application-specific design constraints, propulsion mechanisms, and translational maturity vary across disease domains. Despite this promise, significant cross-cutting challenges remain for clinical translation. These include biocompatibility and long-term safety, achieving precise remote control in vivo, addressing regulatory and ethical hurdles, and establishing scalable, reproducible manufacturing pipelines. A brief overview of progress in these clinical domains and consolidated overview of translational barriers and associated MNR strategies is provided in Table 4.

**Table 4:** Applications of MNRs across Major Biomedical Domains

Category	Oncology	CNS Disorders	GI/IBD	Cardiovascularand Metabolic	Renal (Kidney)	Regenerative Medicine
Major Biological Barrier	Dense tumor microenvironment; poor diffusion; abnormal vasculature	Blood–brain barrier (BBB); limited permeability	Gastric acidity; viscous mucus; epithelial tight junctions	High shear blood flow; hemorrhage risk; systemic toxicity	Glomerular filtration barrier; rapid clearance; podocyte injury; tubular reabsorption limits	Low targeting efficiency; invasive implantation; poor retention
Engineering Strategies	Active navigation; programmable release; theranostics; imaging integration	Biomimetic coatings; receptor-mediated transport; image-guided steering; closed-loop control	Acid-resistant coatings; enzyme-triggered propulsion; biohybrid motors	tPA anchoring; retrievable microswarms; catheter-assisted deployment	Size/charge tuning for filtration; ligand-targeting (podocytes); biodegradable carriers; ultrasound-enhanced retention	3D porous scaffolds; cell-adhesive interfaces; mechanotransductive stimulation
Dominant Actuation Modes	Magnetic; acoustic; chemicallight-triggered	Magnetic; acoustic; AI-guided navigation	Chemical self-propulsion; enzyme-driven; magnetic	Magnetic guidance; ultrasound-mediated	Magnetic; acoustic (ultrasound); flow-assisted navigation	Magnetic; electrical; smart biomaterials
Representative Applications	Tumor-targeted drug delivery; MRI-guided biopsy; hypoxia modulation; deep tumor penetration^108,109^	Neurotherapeutic delivery; BBB penetration; intracranial diagnostics; AI-assisted navigation^110,111^	Mucosal drug delivery; ulcer targeting; microbiome modulation; mucus penetration^112,113^	Targeted thrombolysis; vascular recanalization; clot removal; organ-specific therapy^114,115^	Glomerular-targeted drug delivery; treatment of nephritis; podocyte repair; siRNA/CRISPR delivery; renal fibrosis modulation^109,110^	Stem cell delivery; growth factor transport; tissue repair; neural stimulation^116^

### Oncologic Diseases

MNRs’ ability to actively navigate complex vasculature, penetrate deep tumor tissue, and deliver cytotoxic payloads with high spatial precision makes them uniquely suited for solid-tumor management. Unlike passive nanomedicines, MNRs can traverse intricate vascular networks, overcome dense tumor stroma, and achieve controlled site-specific delivery of therapeutic agents.^107^ Late-stage preclinical studies demonstrate that magnetically guided nanorobots can be steered across organ-scale distances to selectively accumulate at tumor sites, significantly enhancing drug localization and intratumoral penetration while reducing systemic toxicity.^108^ Beyond chemotherapy delivery, MNRs have been explored for tumor hypoxia modulation, precision biopsy enhancement, and localized vaccine administration, and targeted immunotherapeutic deployment.^107^ Their programmability further enables real-time image guidance, theragnostic integration, and controlled release triggered by tumor-specific biochemical or mechanical cues. Although clinical translation is still emerging, current evidence positions MNRs as transformative candidates for minimally invasive, highly targeted oncology interventions.

### Central Nervous System (CNS) Disorders

Magnetically and acoustically actuated MNRs are being developed to overcome the stringent transport restrictions imposed by the blood–brain barrier (BBB), which prevents the entry of more than 98% of small-molecule therapeutics and nearly all biologics.^117^ In contrast to passive nanocarriers, actively propelled MNRs enable field-directed navigation within cerebral vasculature, permitting spatially controlled accumulation at sites of glioblastoma, ischemic, or neuroinflammation. Surface- engineering strategies including biomimetic membrane cloaking and ligand-based receptor-targeting facilitate receptor-mediated transcytosis across the BBB while limiting systemic dispersion.^118^ Complementary micro/nanosystem platforms, including image-guided fiber-based robotic systems, offer localized intraparenchymal delivery with millimeter-scale precision.^111^ By integrating propulsion, targeting, and imaging these systems enabled controlled regional drug deposition within deep brain structures. Although demonstrations remain preclinical, actively navigated MNRs establish a framework for precision neurotherapeutics with enhanced BBB translocation, reduced off-target exposure, and spatially confined CNS intervention.

### Acute and Chronic Kidney Diseases

Micro- and nanorobots have emerged as transformative platforms for targeted renal therapy, offering unprecedented spatiotemporal control over drug delivery, stone fragmentation, and tubular clearance in the complex architecture of the kidney. Catalytic micromotors functionalized with therapeutic agents have demonstrated autonomous navigation through simulated renal tubular environments, exploiting local urea and hydrogen peroxide gradients as endogenous fuel sources to drive propulsion without external power inputs.^108^ Magnetic helical nano swimmers have been shown to traverse the tortuous nephron microenvironment under rotating magnetic field actuation, enabling site-specific payload release at glomerular and tubular targets with minimized off-target accumulation.^111^ Enzyme-powered nanomotors have further been engineered to degrade urinary calculi constituents and biofilm matrices associated with recurrent urinary tract infections, demonstrating efficacy in ex vivo kidney stone models.^108^ Despite these advances, critical translational barriers persist, including renal filtration-mediated clearance of sub-8 nm platforms, immune recognition, potential nephrotoxicity of metallic components, and the challenge of achieving precise closed-loop navigation within the dynamic, high-flow renal vasculature.^111^ Addressing these limitations will require the convergence of biodegradable material design, real-time ultrasound or MRI guidance, and biohybrid actuation strategies to unlock the full therapeutic potential of robotic nanomedicine in nephrology.

### Gastrointestinal (GI) Disorders and Inflammatory Bowel Disease (IBD)

MNRs are being developed to overcome the physicochemical and biological barriers that limit drug delivery in the GI tract, including gastric acidity, mucus viscosity, rapid peristalsis, and epithelial tight junctions.^112^ Magnetically actuated and self-propelled microrobots can be remotely guided through tortuous GI lumens, enabling active mucus penetration and prolonged retention at disease sites.^112^ In IBD, chemically and enzyme-driven MNRs protect encapsulated therapeutics from gastric degradation while selectively accumulating at inflamed colonic mucosa. These platforms enhance local drug bioavailability by navigating toward inflamed tissues rather than relying on passive diffusion or pH-triggered release.^113^ Enzyme-responsive propulsion and biohybrid designs further improve site-specific retention and tissue penetration under dynamic intestinal conditions. Collectively, propulsion-enabled MNRs demonstrate improved spatial control, mucosal penetration, and localized therapeutic deposition in the GI tract. Although current validation remains limited to animal models, these systems lay the groundwork for precision, site-specific intervention in IBD and other GI diseases where conventional oral formulations exhibit limited precision and retention.

### Cardiovascular and Metabolic Diseases

MNRs are being developed for spatially confined thrombolysis in settings where systemic tissue plasminogen activator (tPA) is restricted by hemorrhagic risk and off-target fibrinolysis. Magnetically actuated, tPA-functionalized nanorobots are catheter-delivered and steered to occluding vessels, achieving localized clot dissolution in submillimeter arteries with post-treatment retrieval capability.^115^ This strategy concentrates fibrinolytic activity at the thrombus while minimizing systemic exposure.

Additional field-directed strategies such as magnetically guided MNRs, microbubble-enhanced ultrasound thrombolysis, and electro-thrombectomy have demonstrated improved recanalization under high-shear flow.^114^ Collectively, propulsion-enabled systems demonstrate enhanced thrombus localization and perfusion restoration in vivo, positioning magnetically guided MNRs among the most translational nanorobotic platforms in cardiovascular intervention.

### Regenerative Medicine

MNRs are being explored as mobile platforms for targeted cell delivery and localized regenerative stimulation. Magnetically and electrically responsive micromotors incorporating porous or helical 3D architectures support stem-cell adhesion, transport, and deposition at damaged tissues.^116^ These systems address the low targeting efficiency and invasive delivery requirements of conventional stem-cell therapies by enabling spatially controlled deployment and improved local retention. Beyond cell transport, MNRs can convert external magnetic or electrical fields into localized mechanical or electrical cues that modulate cell proliferation and differentiation.^116^ This propulsion-enabled approach enables minimally invasive stimulation within confined tissue microenvironments, including neural and musculoskeletal tissues. While current progress remains preclinical, these studies highlight MNRs as multifunctional regenerative platforms capable of combining cell carriage, targeted delivery, and mechanotransducive activation.

The current state of MNR development across these five therapeutic domains is summarized in Table 4. Although MNRs are largely in the relatively early stage of development, their activity propelled navigation within cells and tissues provide unique capabilities for precision therapy and localized intervention.

## Artificial Intelligence as a Control Layer for Mechanobiology-Driven MNRs

Artificial intelligence (AI), particularly using machine learning (ML) and deep learning (DL), introduces a computational control layer capable of linking propulsion parameters to biological outcomes under physiological uncertainty. Rather than serving solely as navigation tools, the ML/DL systems enable predictive mapping between actuation inputs (field strength, frequency, torque amplitude) and mechanotransduction responses such as calcium influx, membrane permeabilization, cytoskeletal remodeling.^111,112^ In the context of mechanobiology-driven MNRs, AI facilitates data-driven design and fabrication, adaptive actuation, autonomous navigation and transforms propulsion from an open-loop physical process into a closed-loop, feedback-regulated system.

### AI-Driven Design, Materials Discovery, and Fabrication of MNRs

Traditional design workflows rely on iterative trial-and-error cycles involving conceptual modeling, manual parameter screening, repeated prototyping, and empirical refinement. These processes prolong development timelines and limit scalability. In sharp contrast, ML/DL frameworks offer a data-driven alternative by learning relationships between design parameters and functional performance, enabling predictive optimization and reducing reliance on serial physical prototyping. By mining and extracting relevant features from existing databases, published literature, simulations, and experimental data sets, ML models can predict physicochemical properties and functional behavior, allowing rapid screening of candidate materials before synthesis. Collectively, such approaches replace labor-intensive design pipelines with scalable, simulation-based optimization strategies. Although these applications remain largely at the benchtop or simulation stages, AI-enabled design frameworks establish a foundation for reproducible and translationally reliable MNR fabrication processes.

### Data-Driven Modeling of Propulsion-Biology Coupling

MNR behavior emerges from coupled nonlinear interactions between fields, fluid environments, structure mechanics, and several biological interfaces. Analytical models often fail to capture key resources of variability, including tissue-dependent viscosity, protein corona formation, and cell-specific mechanosensitivity.^114^ ML-based modeling can integrate diverse data streams, including imaging-derived trajectories, fluid dynamic simulations, cellular calcium response profiles, and force-deformation measurements.^115^ Supervised learning models trained on physics-based simulation libraries predict propulsion velocity, torque response, and deformation stability.^112^

Reinforcement learning agents, such as the example depicted in Figure 7, optimize reward functions encoding target proximity, collision avoidance, and energy efficiency.^113^ These agents outperform static planners in complex geometries. Gaussian process regression and Bayesian optimization further support efficient parameter tuning under limited sampling conditions.^114^ However, reliability of these models depends critically on training data representativeness. Domain shift between in vitro calibration and in vivo deployment remains a key unresolved challenge.^115^

**Figure 7:**
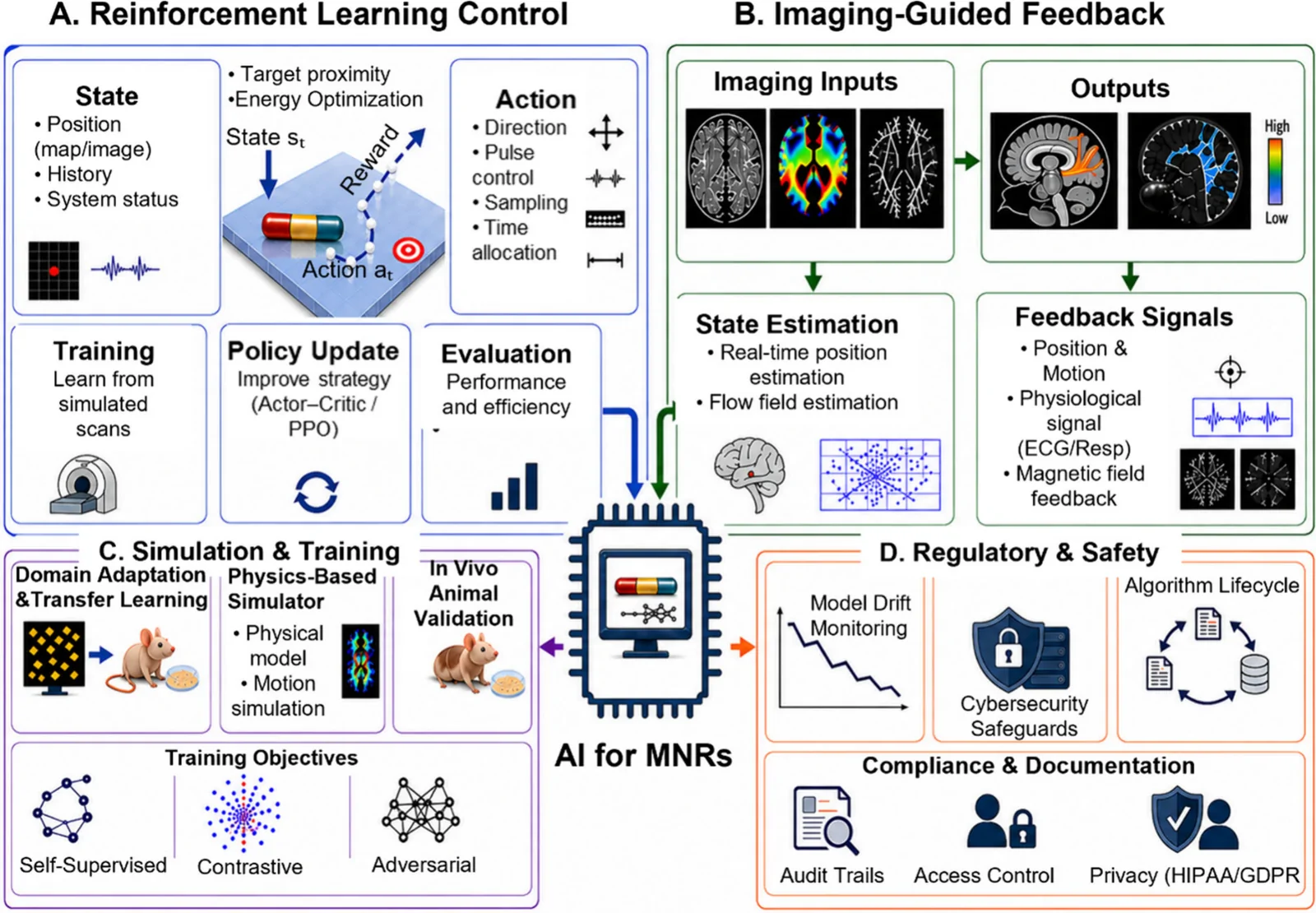
AI for mechanobiology-driven MNRs. (A) An example of reinforcement learning is shown, in which propulsion is optimized through interactive state-action-reward policy updates. (B) Real-time imaging provides continuous feedback, while simulation-to-in vivo validation supports domain adaptation (C). (D) In parallel, regulatory modules address model drift, cybersecurity vulnerabilities, and overall algorithmic governance. Note: this figure is intended solely for conceptual visualization.

AI-guided MNRs require an integrated data and training pipeline that spans simulation, in vitro, and in vivo environments, each contributing uniquely to model development and validation. Simulation data sets, typically derived from physics-based Multiphysics models (e.g., magnetics, acoustics, microfluidics) or high-throughput synthetic environments, provide the large, diverse, and fully labeled state–action–reward trajectories needed for reinforcement learning (RL) policies and control-oriented surrogate models To ensure robust real-world transfer, these data sets must incorporate domain randomization factors such as viscosity ranges, flow perturbations, Brownian noise, and variability in anatomical geometry. In vitro data sets are generated using microfluidic phantoms, tissue-mimicking gels, MRI/US/PA imaging recordings, and high-resolution optical microscopy. These data sets supply ground-truth trajectories, segmentation masks, detection/tracking frames, and closed-loop demonstrations that refine simulation-trained controllers through supervised fine-tuning or offline RL.

In vivo data sets which are far smaller and substantially more difficult to annotate, are reserved for late-stage policy validation. These studies typically use real-time MRI, ultrasound, or optoacoustic tracking, coupled with objective end points such as navigation accuracy, target-hit rate, retention time, biodistribution, and safety. Training pipelines generally follow a structured sequence: (i) large-scale RL/DL pretraining in simulation, (ii) sim-to-real adaptation via domain randomization, privileged/distilled policies, or system-identification loops, (iii) in vitro fine-tuning under realistic imaging noise and fluid dynamics, and (iv) constrained in vivo testing with safety wrappers, confidence-based action gating, or model-predictive safety filters. Validation strategies must employ multitier benchmarks, including simulation benchmarks (navigation accuracy and sample-efficiency curves, and energy cost); in vitro benchmarks (tracking precision under imaging artifacts, obstacle-avoidance success, and swarm-cohesion metrics); and in vivo benchmarks (tissue-scale trajectory fidelity, target-delivery rate, immunological tolerance, and robustness to failure modes). Together, these data, training, and validation requirements establish a reproducible, regulator-aligned pipeline for deploying AI-enabled MNRs in clinically meaningful settings (Figure 7)

### Imaging-Guided Feedback and Closed-Loop Control

High-resolution tracking remains a critical bottleneck for the clinical translation of MNRs. Examples of imaging-based tracking and design/fabrication for AI-enabled MNRs are listed in Table 5. Deep neural networks (DNNs) trained on imaging data sets from modalities such as ultrasound, MRI, fluorescence microscopy provide dynamic feedback on MNR position and local biological responses.^109–111^

**Table 5:** Examples of Imaging-Based Tracking and Design/Fabrication for AI-MNRs

Platform/Modality	ML/RL Method	Task (context)	Key Outcome
MRI-powered magnetic microrobots	CNN + particle filter	3D tracking from 2D MR slices (in vitro/ex vio)	±1.43 mm 3D accuracy; 60% improvement vs prior MRI tracking.^116^
Ultrasound imaging of microrobots	DL detectors (ResNeSt-269, ConvNeXt, YOLOv11/12, U-Net)	Benchmark detection/tracking for US microrobotics	ResNeSt-269 best accuracy; YOLO fastest real time.^117^
USMicroMagSet data set (magnetic microrobots)	Curated DL data set + baselines	∼40k annotated US frames; single and swarm agents	Enables reproducible training/benchmarking for US tracking.^118^
US color-flow mapping (pseudo-Doppler)	Signal processing + DL pipeline	Simultaneous localization and activation of US microrobots	Real-time visualization of 60–80 μm robots via color-flow mapping.^119^
Optoacoustic (photoacoustic) imaging of magnetic microrobots	DL-assisted tracking (NIR enhancement)	Real-time 3D tracking/manipulation in mouse brain vasculature	Volumetric tracking of 5–20 μm microrobots in vivo.^120^
μ-LPBF (microscale laser powder-bed fusion)	Supervised ML + SHAP; multiobjective optimization	Predict/optimize track morphology (spray/laser parameters)	R^2^ ≥ 0.8; submicrometer roughness (Ra < 0.5 μm) for printable parts.^121^
Design-to-navigation mapping	Review of ML across pipeline	Curates exemplars and best practices	Guidance for data-driven microrobot design and evaluation.^122^

Generative adversarial networks (GANs) further augment limited experimental microscopy data sets by producing synthetic images that capture variations in object orientation, illumination, and imaging parameters. Models trained on this combined simulated and experimental data set can then be validated on previously unseen real images, achieving accurate estimation of both translational and rotational pose components. AI-based segmentation and tracking algorithms can quantify trajectory deviations, flow-field changes, and intracellular signaling events. Closed-loop systems integrate this sensor feedback to adjust acoustic frequency, magnetic field orientation, actuation amplitude, pulse duration.^111–113^ Such adaptive control permits force modulation within predefined biological safety windows, minimizing unintended tissue damage.^111^ Importantly, closed-loop control aligns with mechanobiological calibration by keeping propulsion-induced stresses within cellular activation thresholds while avoiding mechanical rupture.

Particularly, for MNR-induced mechanotransduction studies elaborated in Figure 2, hybrid multimodal CNN–GNN (graph neural network) frameworks provide a powerful paradigm for integrating multichannel immunofluorescent (IF) imaging with spatial, molecular, and experimental metadata.^112^ In this approach, CNNs are first employed to extract rich, hierarchical features from IF images, where multiple fluorescence channels capture mechanosensitive markers such as integrins, focal adhesion proteins, cytoskeletal components, and nuclear mechanotransducers (e.g., YAP/TAZ, ERK). CNNs excel at quantifying subcellular morphology, protein localization, and intensity patterns directly from pixel-level data, enabling robust characterization of local cellular responses to MNR applied forces. As illustrated in the Figure S1, these learned image features are then integrated into GNNs, where individual cells are represented as nodes enriched with CNN-derived embeddings and molecular measurements, while edges encode spatial proximity, cell–cell contacts, or MNR-mediated force coupling. This graph-based representation allows explicit modeling of how mechanical cues propagate across cellular neighborhoods, capturing collective and emergent behaviors that are central to mechanotransduction but difficult to resolve with image only models. By fusing IF image features with spatial relationships and contextual metadata (such as actuation parameters, time points, or cell type), multimodal CNN–GNN architectures enable interpretable, pathway level inference of mechanotransducive signaling, linking MNR induced physical perturbations to coordinated activation of integrin–FAK, YAP/TAZ, and ERK pathways at the tissue or microenvironment scale.

### AI for Swarm Coordination

Swarm-based MNR systems exhibit emergent collective dynamics that require specialized coordination strategies. Examples of AI-driven swarm control and swarm-intelligence-enabled MNR are shown in Table 6. Multiagent reinforcement learning (MARL) enables distributed coordination for density regulation, cooperative transport, and obstacle avoidance. Convolutional neural-network-based state are increasingly used to infer swarm configurations and update global magnetic-field inputs in real-time.^123^ To-date, most demonstrations of AI-enabled swarm control remain limited to simulation studies or small-scale benchtop experiments, with large-scale in vivo studies lacking. Collective aggregation also introduces specific safety concerns, including the potential for transient or sustained vascular obstruction. Therefore, safety assessment must explicitly evaluate emergent behavior under physiological flow conditions to ensure predictable and clinically acceptable swarm dynamics.

**Table 6:** Examples of Control and Swarm Intelligence for AI-MNRs

Platform/Actuation	ML/RL Method	Task	Key Outcome
Magnetic microrobot (EM coils)	RL (gradual training; sim-to-real)	Autonomous 3D positional control via direct coil-current policy	RL outperformed PID; higher positioning accuracy/efficiency.^126^
Magnetic helical microrobot in dynamic flows	SAC (RL) + transformer encoder	Energy-efficient, obstacle-free navigation in time-varying flows	∼15% lower energy; robust obstacle avoidance (sim + hardware).^115^
Ultrasound-actuated microrobot	Model-based RL (imagined rollouts)	Autonomous ultrasound navigation with image feedback; sim pretrain + fast fine-tune	∼90% success after ∼1 h; adapts to new channels.^115^
Ultrasound microswarms (microbubbles)	Deep RL + detection/tracking	Autonomous acoustic navigation and trajectory following	RL-driven ultrasound manipulation with >100k images for training.^127^
Magnetic spiral microrobot under US/PA imaging	DL detector in closed loop (GPU beamforming)	Real-time control under dual-mode imaging	100 ms loop; PA-guided DL reduces missed detections vs US alone.^128^
Optically actuated microrobots (≤200 agents)	Multiagent RL with counterfactual rewards	Collective transport of large cargo; ant-like strategies	Robust, scalable; resilient to failures and noise.^120^
Magnetic swarms	PPO + Transformer; domain randomization	Autonomous swarm navigation/obstacle avoidance (POMDP)	Maintains pattern; tracks moving targets; sim-to-real strategy.^127^

### Challenges of AI-Enabled MNRs: Translational, Regulatory, and Ethical Considerations

In addition to the general limitations of nanosystems for passive nanomedicines as described previously in individual sections, AI-enabled and actively actuated MNRs introduce additional translational and regulatory risks, which are schematically shown in Figure 8. Persistent magnetic or metallic components may accumulate within tissues, necessitating comprehensive long-term biodistribution and clearance studies.^115,124,125^ Actively propelled systems may generate localized mechanical stresses capable of damaging sensitive or inflamed tissues, if not properly constrained. Remote actuation introduces an additional layer of cybersecurity vulnerability. Developers must therefore implement encrypted command architectures, enforce predefined actuation limits, and incorporate reliable fail-safe deactivation mechanisms.

**Figure 8:**
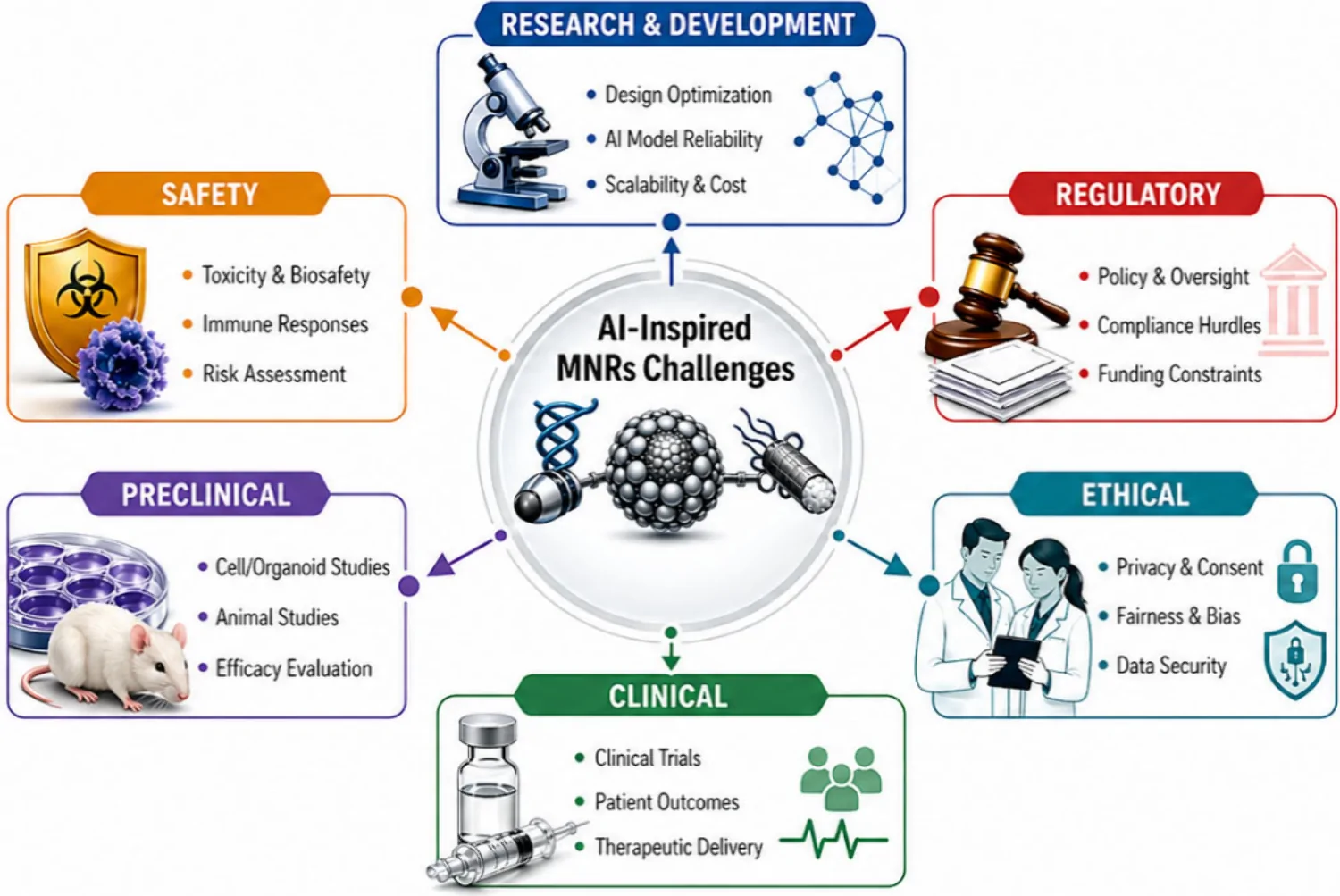
Translational challenges of AI-inspired micro/nanorobots (MNRs). Integrated schematic summarizing key barriers across development, safety, regulation, ethics, preclinical validation, and clinical translation, highlighting the interconnected requirements for effective biomedical deployment. Note: this figure is intended solely for conceptual visualization.

Whenever possible, retrievable, dissolvable, or biodegradable device architecture should be prioritized to minimize long-term retention risks. AI-enabled MNRs equipped with internal biosensing capabilities pose further challenges, as they collect highly sensitive physiological data. Such systems require dynamic consent models, strict data minimization, secure transmission protocols, and clearly defined retention and deletion policies.^17,129,130^ Regulatory evaluation for these platforms will likely draw from multiple frameworks, spanning device regulations, combination-product pathways, and software-as-a-medical-device requirements. Approval will require evidence of algorithm stability, continuous monitoring for model drift, robust cybersecurity auditing, and standardized simulation-to-in vivo validation pipelines. These elements must be established before clinical deployment can be pursued. Key considerations for translational MNRs are graphically shown in Figure 8.

## Concluding Remarks and Future Directions

Magnetically, acoustic, catalytically, and optically propelled microbots (MNRs) extend nanomedicine beyond passive transport toward actively actuated, force-enabled therapeutic platforms. Each propulsion modality produces distinct mechanical signatures capable of modulating membrane permeability, cytoskeletal organization, and microenvironmental dynamics in unique ways. By coupling propulsion-induced mechanical stimuli to cellular mechanotransduction pathways, MNRs establish a programmable interface between physical energy and biological responses. This mechanobiological paradigm differentiates MNRs from conventional nanocarriers and defines their translational potential. As the field advances, several research priorities will shape progress toward clinical implementation.

Key future research priorities include the following. (i) Developing robust, reproducible methods for industrial-scale production of MNRs that minimize batch-to-batch variability, especially for complex hybrid and biohybrid platforms. This will require standardized protocols for critical quality attributes such as particle size, polydispersity index, and encapsulation efficiency. (ii) Conducting comprehensive investigations into long-term biocompatibility, biodistribution, and potential toxicity of MNRs in vivo, along with creation of reliable strategies for retrieval, deactivation, or biodegradation post-therapy. (iii) Optimizing multimodal propulsion mechanisms to achieve precise, adaptable control in complex biological environments; integrating real-time imaging and closed-loop feedback systems for accurate tracking and navigation; and exploring swarm intelligence or cooperative behaviors for collective targeting and therapeutic action. (iv) Integration with ML and DL framework to enable adaptive control, autonomous decision-making, real-time navigation, and patient-specific navigation within heterogeneous physiological environments. Together, these advancements will position MNRs as controllable mechanobiological systems rather than passive delivery vehicles.

Clinical translation will require scalable manufacturing pipelines, quantitative definition of mechanobiological safety thresholds, standardized in vivo testing protocols, and tiered regulatory frameworks that address both active physical actuation and AI-enabled control. Long-term biodistribution, tissue-level force tolerances, and cybersecurity safeguards for autonomous or semiautonomous systems must be rigorously defined.

In summary, mechanobiology-driven, intelligent, adaptable, and clinically integrated MNRs represent a transition toward force-active nanomedicine, in which propulsion-generated mechanical inputs are deliberately engineered to elicit targeted biological outcomes. Ultimately, the clinical impact of these systems will hinge on disciplined engineering, quantitative validation, and early incorporation of regulatory considerations into their design.

## Supplementary Material



## Notes

Propulsion mode characteristics and example of multispectral image analysis for mechanotransduction studies using a hybrid CNN/GNN approach (PDF)

## Abbreviations

AAO: anodic aluminum oxide
ATP: adenosine triphosphate
CNN: convolutional neural network
CQAs: critical quality attributes
DL: deep learning
DNN: deep neural network
ECM: extracellular matrix
eNOS: endothelial nitric oxide synthase
FAK: focal adhesion kinase
GAN: generative adversarial network
IBD: inflammatory bowel disease
μ-LPBF: microlaser powder bed fusion
MAPK: mitogen-activated protein kinase
MARL: multiagent reinforcement learning
MRI: magnetic resonance imaging
MNRs: micro/nanorobots
NFAT: nuclear factor of activated T cells
NIR: near-infrared
PA: photoacoustic
PAMAM: poly­(amidoamine)
PBAE: poly­(β-amino ester)
PEG: polyethylene glycol
PID: proportional–integral–derivative
PI3K–Akt: phosphoinositide 3-kinase/protein kinase B
PLGA: poly­(lactic-*co*-glycolic acid)
POD: proper orthogonal decomposition
PPO: proximal policy optimization
RES: reticuloendothelial system
RL: reinforcement learning
ROS: reactive oxygen species
SAC: soft actor-critic
SAMs: self-assembled monolayers
SHAP: Shapley additive explanations
tPA: tissue plasminogen activator
TRPV4: transient receptor potential vanilloid 4
TEAD: TEA domain transcription factor
YAP: yes-associated protein
YOLO: you only look once

## References

[1] Azar, A. T. ; Madian, A. ; Ibrahim, H. ; Taha, M. A. ; Mohamed, N. A. ; Fathy, Z. ; AboAlNaga, B. M. Medical Nanorobots: Design, Applications and Future Challenges. In Control Systems Design of Bio-Robotics and Bio-mechatronics with Advanced Applications; Elsevier, 2020; pp 329–394. 10.1016/B978-0-12-817463-0.00011-3.

[2] Dutta, D. ; Sailapu, S. K. Biomedical Applications of Nanobots. In Intelligent Nanomaterials for Drug Delivery Applications; Elsevier, 2020; pp 179–195. 10.1016/B978-0-12-817830-0.00010-2.

[3] Zhang L., Du Y., Wang W., Gao R., Dong Y., Wang Q., Tong F. (2025). Biomedical Treatment Nanomotors Based on the Intestinal Axis: Current
Challenges. ACS Appl. Bio Mater..

[4] Li M., Hu X., Zhao Y., Jiao N. (2023). An Overview of Recent Progress in
Micro/Nanorobots for Biomedical Applications. Adv. Mater. Technol..

[5] Li M., Xi N., Wang Y., Liu L. (2021). Progress in Nanorobotics for Advancing
Biomedicine. IEEE Trans. Biomed. Eng..

[6] Zhao H., Wang Y., Bao L., Chen C. (2022). Engineering Nano–Bio
Interfaces from Nanomaterials to Nanomedicines. Acc. Mater. Res..

[7] Martino F., Perestrelo A. R., Vinarský V., Pagliari S., Forte G. (2018). Cellular Mechanotransduction:
From Tension to Function. Front. Physiol..

[8] Discher D. E., Janmey P., Wang Y. (2005). Tissue Cells
Feel and Respond to
the Stiffness of Their Substrate. Science.

[9] O’Neill G. M., Zhu C., Kim D.-H., Shin J. (2025). Mechanomedicine: Translating Mechanical
Forces into Therapeutic Strategies. APL Bioeng..

[10] Liu Z., Chen G., Jo M.-S., Vogel V., Chen J., Rogers J. A., Li S. (2026). Mechanomedicine. Nat. Rev. Bioeng..

[11] Kumari A., Veena S. M., Luha R., Tijore A. (2023). Mechanobiological Strategies
to Augment Cancer Treatment. ACS Omega.

[12] Li Z., Zhu Y., Zeng H., Wang C., Xu C., Wang Q., Wang H., Li S., Chen J., Xiao C., Yang X., Li Z. (2023). Mechano-Boosting Nanomedicine Antitumour
Efficacy by Blocking the Reticuloendothelial System with Stiff Nanogels. Nat. Commun..

[13] Yang Z., Zhao Y., Zhang X., Huang L., Wang K., Sun J., Chen N., Yin W., Chen S., Zhi H., Xue L., An L., Li R., Dong H., Xu J., Li Y., Li Y. (2024). Nano-Mechanical
Immunoengineering: Nanoparticle Elasticity
Reprograms Tumor-Associated Macrophages via Piezo1. ACS Nano.

[14] Ahmed S., Wang W., Bai L., Gentekos D. T., Hoyos M., Mallouk T. E. (2016). Density and Shape
Effects in the Acoustic Propulsion
of Bimetallic Nanorod Motors. ACS Nano.

[15] Purcell E. M. (1977). Life at
Low Reynolds Number. Am. J. Phys..

[16] Fournier-Bidoz S., Arsenault A. C., Manners I., Ozin G. A. (2005). Synthetic Self-Propelled
Nanorotors. Chem. Commun..

[17] Ju X., Chen C., Oral C. M., Sevim S., Golestanian R., Sun M., Bouzari N., Lin X., Urso M., Nam J. S., Cho Y., Peng X., Landers F. C., Yang S., Adibi A., Taz N., Wittkowski R., Ahmed D., Wang W., Magdanz V., Medina-Sánchez M., Guix M., Bari N., Behkam B., Kapral R., Huang Y., Tang J., Wang B., Morozov K., Leshansky A., Abbasi S. A., Choi H., Ghosh S., Borges Fernandes B., Battaglia G., Fischer P., Ghosh A., Jurado Sánchez B., Escarpa A., Martinet Q., Palacci J., Lauga E., Moran J., Ramos-Docampo M. A., Städler B., Herrera Restrepo R. S., Yossifon G., Nicholas J. D., Ignés-Mullol J., Puigmartí-Luis J., Liu Y., Zarzar L. D., Shields C. W., Li L., Li S., Ma X., Gracias D. H., Velev O., Sánchez S., Esplandiu M. J., Simmchen J., Lobosco A., Misra S., Wu Z., Li J., Kuhn A., Nourhani A., Maric T., Xiong Z., Aghakhani A., Mei Y., Tu Y., Peng F., Diller E., Sakar M. S., Sen A., Law J., Sun Y., Pena-Francesch A., Villa K., Li H., Fan D. E., Liang K., Huang T. J., Chen X.-Z., Tang S., Zhang X., Cui J., Wang H., Gao W., Kumar Bandari V., Schmidt O. G., Wu X., Guan J., Sitti M., Nelson B. J., Pané S., Zhang L., Shahsavan H., He Q., Kim I.-D., Wang J., Pumera M. (2025). Technology Roadmap of Micro/Nanorobots. ACS Nano.

[18] Ibele M. E., Lammert P. E., Crespi V. H., Sen A. (2010). Emergent, Collective
Oscillations of Self-Mobile Particles and Patterned Surfaces under
Redox Conditions. ACS Nano.

[19] Kar A., Chiang T.-Y., Ortiz
Rivera I., Sen A., Velegol D. (2015). Enhanced Transport
into and out of Dead-End Pores. ACS Nano.

[20] Unruh A., Brooks A. M., Aranson I. S., Sen A. (2023). Programming Motion
of Platinum Microparticles: From Linear to Orbital. ACS Appl. Eng. Mater..

[21] Gentile K., Somasundar A., Bhide A., Sen A. (2020). Chemically Powered
Synthetic “Living” Systems. Chem..

[22] Xiao B. (2024). Mechanisms
of Mechanotransduction and Physiological Roles of PIEZO Channels. Nat. Rev. Mol. Cell Biol..

[23] Verkest C., Schaefer I., Nees T. A., Wang N., Jegelka J. M., Taberner F. J., Lechner S. G. (2022). Intrinsically
Disordered Intracellular
Domains Control Key Features of the Mechanically-Gated Ion Channel
PIEZO2. Nat. Commun..

[24] Yao M., Tijore A., Cheng D., Li J. V., Hariharan A., Martinac B., Tran Van
Nhieu G., Cox C. D., Sheetz M. (2022). Force- and
Cell State–Dependent Recruitment of Piezo1 Drives Focal Adhesion
Dynamics and Calcium Entry. Sci. Adv..

[25] Jetta D., Gottlieb P. A., Verma D., Sachs F., Hua S. Z. (2019). Shear Stress-Induced
Nuclear Shrinkage through Activation of Piezo1 Channels in Epithelial
Cells. J. Cell Sci..

[26] Bera D., Kuiry S. C., Seal S. (2004). Synthesis
of Nanostructured Materials
Using Template-Assisted Electrodeposition. JOM.

[27] Alghazali K. M., Newby S. D., Nima Z. A., Hamzah R. N., Watanabe F., Bourdo S. E., Masi T. J., Stephenson S. M., Anderson D. E., Dhar M. S., Biris A. S. (2017). Functionalized Gold
Nanorod Nanocomposite System to Modulate Differentiation of Human
Mesenchymal Stem Cells into Neural-like Progenitors. Sci. Rep..

[28] He T., Yang Y., Chen X.-B. (2023). Preparation, Stimulus–Response
Mechanisms and Applications of Micro/Nanorobots. Micromachines.

[29] Selva
Sharma A., Lee N. Y. (2024). Recent Advances in Micro- and Nanorobot-Assisted
Colorimetric and Fluorescence Platforms for Biosensing Applications. Micromachines.

[30] Xu D., Wang Y., Liang C., You Y., Sanchez S., Ma X. (2020). Self-Propelled Micro/Nanomotors for On-Demand Biomedical Cargo Transportation. Small.

[31] Macchione M., Biglione C., Strumia M. (2018). Design, Synthesis and Architectures
of Hybrid Nanomaterials for Therapy and Diagnosis Applications. Polymers.

[32] Song W., Li L., Liu X., Zhu Y., Yu S., Wang H., Wang L. (2024). Hydrogel Microrobots for Biomedical Applications. Front. Chem..

[33] Han H., Ma X., Deng W., Zhang J., Tang S., Pak O. S., Zhu L., Criado-Hidalgo E., Gong C., Karshalev E., Yoo J., You M., Liu A., Wang C., Shen H. K., Patel P. N., Hays C. L., Gunnarson P. J., Li L., Zhang Y., Dabiri J. O., Wang L. V., Shapiro M. G., Wu D., Zhou Q., Greer J. R., Gao W. (2024). Imaging-Guided Bioresorbable
Acoustic Hydrogel Microrobots. Sci. Robot..

[34] Zhang H., Yang M., Kim S. H., Li I. T. S. (2025). Integrin
Force
Loading Rate in Mechanobiology: From Model to Molecular Measurement. QRB Discovery.

[35] Kechagia J. Z., Ivaska J., Roca-Cusachs P. (2019). Integrins as Biomechanical Sensors
of the Microenvironment. Nat. Rev. Mol. Cell
Biol..

[36] Gomez A., Muzzio N., Dudek A., Santi A., Redondo C., Zurbano R., Morales R., Romero G. (2023). Elucidating
Mechanotransduction
Processes During Magnetomechanical Neuromodulation Mediated by Magnetic
Nanodiscs. Cell. Mol. Bioeng..

[37] Henstock J. R., Rotherham M., Rashidi H., Shakesheff K. M., El Haj A. J. (2014). Remotely Activated
Mechanotransduction via Magnetic
Nanoparticles Promotes Mineralization Synergistically With Bone Morphogenetic
Protein 2: Applications for Injectable Cell Therapy. Stem Cells Transl. Med..

[38] Sheetz M. P. (2001). Cell Control
by Membrane–Cytoskeleton Adhesion. Nat.
Rev. Mol. Cell Biol..

[39] Isomursu A., Lerche M., Taskinen M. E., Ivaska J., Peuhu E. (2019). Integrin Signaling
and Mechanotransduction in Regulation of Somatic Stem Cells. Exp. Cell Res..

[40] Discher D. E., Janmey P., Wang Y. (2005). Tissue Cells
Feel and Respond to
the Stiffness of Their Substrate. Science.

[41] Roein-Peikar M., Xu Q., Wang X., Ha T. (2016). Ultrasensitivity of Cell Adhesion
to the Presence of Mechanically Strong Ligands. Phys. Rev. X.

[42] Engler A. J., Sen S., Sweeney H. L., Discher D. E. (2006). Matrix
Elasticity Directs Stem Cell
Lineage Specification. Cell.

[43] Davies P. F. (2009). Hemodynamic
Shear Stress and the Endothelium in Cardiovascular Pathophysiology. Nat. Clin. Pract. Cardiovasc. Med..

[44] Chen S., Fan D. E., Fischer P., Ghosh A., Göpfrich K., Golestanian R., Hess H., Ma X., Nelson B. J., Patiño
Padial T., Tang J., Villa K., Wang W., Zhang L., Sen A., Sánchez S. (2025). A Roadmap
for Next-Generation Nanomotors. Nat. Nanotechnol..

[45] Hajiali H., Rotherham M., El Haj A. J. (2024). Remote Activation of Mechanotransduction
via Integrin Alpha-5 via Aptamer-Conjugated Magnetic Nanoparticles
Promotes Osteogenesis. Pharmaceutics.

[46] Abdel
Fattah A. R., Kolaitis N., Van Daele K., Daza B., Rustandi A. G., Ranga A. (2023). Targeted Mechanical
Stimulation via Magnetic Nanoparticles Guides in Vitro Tissue Development. Nat. Commun..

[47] Tajik A., Zhang Y., Wei F., Sun J., Jia Q., Zhou W., Singh R., Khanna N., Belmont A. S., Wang N. (2016). Transcription Upregulation via Force-Induced Direct Stretching of
Chromatin. Nat. Mater..

[48] Bryniarska-Kubiak N., Kubiak A., Basta-Kaim A. (2023). Mechanotransductive
Receptor Piezo1
as a Promising Target in theTreatment of Neurological Diseases. Curr. Neuropharmacol..

[49] Afsari T., Nowlin K., Weissing C., Ahmed S. (2024). Nanorobotic System
with Fine Control over Multiple Modes of Motion. ACS Appl. Nano Mater..

[50] McMahon H. T., Gallop J. L. (2005). Membrane Curvature
and Mechanisms of Dynamic Cell Membrane
Remodelling. Nature.

[51] Lewis A. H., Grandl J. (2015). Mechanical Sensitivity
of Piezo1 Ion Channels Can Be
Tuned by Cellular Membrane Tension. eLife.

[52] Mamidi N., Franco De Silva F., Orash Mahmoudsalehi A. (2025). Advanced Disease Therapeutics Using
Engineered Living Drug Delivery Systems. Nanoscale.

[53] Butcher D.
T., Alliston T., Weaver V. M. (2009). A Tense Situation: Forcing Tumour
Progression. Nat. Rev. Cancer.

[54] Champion J. A., Mitragotri S. (2006). Role of Target
Geometry in Phagocytosis. Proc. Natl. Acad.
Sci. U. S. A..

[55] Rashid F., Kabbo S. A., Wang N. (2024). Mechanomemory
of Nucleoplasm and
RNA Polymerase II after Chromatin Stretching by a Microinjected Magnetic
Nanoparticle Force. Cell Rep..

[56] Terstappen G. C., Meyer A. H., Bell R. D., Zhang W. (2021). Strategies for Delivering
Therapeutics across the Blood–Brain Barrier. Nat. Rev. Drug Discovery.

[57] Zhao P., Wu T., Tian Y., You J., Cui X. (2024). Recent Advances of
Focused Ultrasound Induced Blood-Brain Barrier Opening for Clinical
Applications of Neurodegenerative Diseases. Adv. Drug Delivery Rev..

[58] Zha S., Liu H., Li H., Li H., Wong K.-L., All A. H. (2024). Functionalized
Nanomaterials Capable of Crossing the Blood–Brain Barrier. ACS Nano.

[59] Chen Y., Sun H., Li Y., Han X., Yang Y., Chen Z., Zhao X., Qian Y., Liu X., Zhou F., Bai J., Qiao Y. (2025). Magnetic Nanomaterials
for Hyperthermia-Based Therapy
and Controlled Drug Delivery. Bioact. Mater..

[60] Chen J., Yuan M., Madison C. A., Eitan S., Wang Y. (2022). Blood-Brain
Barrier Crossing Using Magnetic Stimulated Nanoparticles. J. Controlled Release.

[61] Patrick P. S., Stuckey D. J., Zhu H., Kalber T. L., Iftikhar H., Southern P., Bear J. C., Lythgoe M. F., Hattersley S. R., Pankhurst Q. A. (2024). Improved Tumour Delivery of Iron
Oxide Nanoparticles
for Magnetic Hyperthermia Therapy of Melanoma *via* Ultrasound Guidance and^111^ In SPECT Quantification. Nanoscale.

[62] Thomsen L. B., Thomsen M. S., Moos T. (2015). Targeted Drug
Delivery to the Brain
Using Magnetic Nanoparticles. Ther. Delivery.

[63] Hortelão A. C., Patiño T., Perez-Jiménez A., Blanco À., Sánchez S. (2018). Enzyme-Powered
Nanobots Enhance Anticancer
Drug Delivery. Adv. Funct. Mater..

[64] Guix M., Meyer A. K., Koch B., Schmidt O. G. (2016). Carbonate-Based
Janus Micromotors Moving in Ultra-Light Acidic Environment Generated
by HeLa Cells in Situ. Sci. Rep..

[65] Safdar M., Khan S. U., Janis J. (2018). Progress toward
Catalytic Micro-
and Nanomotors for Biomedical and Environmental Applications. Adv. Mater..

[66] Xu L., Mou F., Gong H., Luo M., Guan J. (2017). Light-Driven Micro/Nanomotors:
From Fundamentals to Applications. Chem. Soc.
Rev..

[67] Abid J.-P., Frigoli M., Pansu R., Szeftel J., Zyss J., Larpent C., Brasselet S. (2011). Light-Driven Directed Motion of Azobenzene-Coated
Polymer Nanoparticles in an Aqueous Medium. Langmuir.

[68] Liang Z., Teal D., Fan D. (2019). Light Programmable Micro/Nanomotors
with Optically Tunable in-Phase Electric Polarization. Nat. Commun..

[69] Ferreira V. R. A., Azenha M. A. (2024). Recent Advances in Light-Driven Semiconductor-Based
Micro/Nanomotors: Optimization Strategies and Emerging Applications. Molecules.

[70] Du Z., Chen G., Li Y., Zheng N., Cheng J.-X., Yang C. (2024). Photoacoustic: A Versatile Nongenetic Method for High-Precision Neuromodulation. Acc. Chem. Res..

[71] Wang W., Duan W., Ahmed S., Mallouk T. E., Sen A. (2013). Small Power:
Autonomous Nano- and Micromotors Propelled by Self-Generated Gradients. Nano Today.

[72] Bruus H. (2012). Acoustofluidics
7: The Acoustic Radiation Force on Small Particles. Lab. Chip.

[73] Brunton S. L., Noack B. R., Koumoutsakos P. (2020). Machine Learning for Fluid Mechanics. Annu. Rev. Fluid Mech..

[74] Andrade M. A. B., Pérez N., Adamowski J. C. (2015). Particle Manipulation by a Non-Resonant
Acoustic Levitator. Appl. Phys. Lett..

[75] Durham P.
G., Butnariu A., Alghorazi R., Pinton G., Krishna V., Dayton P. A. (2024). Current
Clinical Investigations of Focused Ultrasound
Blood-Brain Barrier Disruption: A Review. Neurotherapeutics.

[76] Del
Campo Fonseca A., Glück C., Droux J., Ferry Y., Frei C., Wegener S., Weber B., El Amki M., Ahmed D. (2023). Ultrasound Trapping and Navigation of Microrobots in the Mouse Brain
Vasculature. Nat. Commun..

[77] Uygun M., Jurado-Sánchez B., Uygun D. A., Singh V. V., Zhang L., Wang J. (2017). Ultrasound-Propelled
Nanowire Motors
Enhance Asparaginase Enzymatic Activity against Cancer Cells. Nanoscale.

[78] Zhou H., Mayorga-Martinez C. C., Pané S., Zhang L., Pumera M. (2021). Magnetically
Driven Micro and Nanorobots. Chem. Rev..

[79] Matos A. M., Gonçalves A. I., El Haj A. J., Gomes M. E. (2020). Magnetic Biomaterials
and Nano-Instructive Tools as Mediators of Tendon Mechanotransduction. Nanoscale Adv..

[80] Ablay G., Böyük M., İçöz K. (2019). Design, Modeling, and
Control of a Horizontal Magnetic Micromanipulator. Trans. Inst. Meas. Control.

[81] Liu Y., Ge D., Cong J., Piao H.-G., Huang X., Xu Y., Lu G., Pan L., Liu M. (2018). Magnetically Powered Annelid-Worm-Like
Microswimmers. Small.

[82] Liu J. F., Jang B., Issadore D., Tsourkas A. (2019). Use of Magnetic Fields
and Nanoparticles to Trigger Drug Release and Improve Tumor Targeting. WIREs Nanomedicine Nanobiotechnology.

[83] Qiao S., Ouyang H., Zheng X., Qi C., Ma L. (2023). Magnetically
Actuated Hydrogel-Based Capsule Microrobots for Intravascular Targeted
Drug Delivery. J. Mater. Chem. B.

[84] Paxton W.
F., Kistler K. C., Olmeda C. C., Sen A., St. Angelo S. K., Cao Y., Mallouk T. E., Lammert P. E., Crespi V. H. (2004). Catalytic nanomotors:
autonomous movement of striped nanorods. J.
Am. Chem. Soc..

[85] Gao W., Dong R., Thamphiwatana S., Li J., Gao W., Zhang L., Wang J. (2015). Artificial Micromotors in the Mouse’s
Stomach: A Step toward *in Vivo* Use of Synthetic Motors. ACS Nano.

[86] Ebbens S. J., Gregory D. A. (2018). Catalytic Janus Colloids: Controlling Trajectories
of Chemical Microswimmers. Acc. Chem. Res..

[87] Baraban L., Streubel R., Makarov D., Han L., Karnaushenko D., Schmidt O. G., Cuniberti G. (2013). Fuel-Free Locomotion of Janus Motors:
Magnetically Induced Thermophoresis. ACS Nano.

[88] Grier D. G. (2003). A Revolution
in Optical Manipulation. Nature.

[89] Ferreira V. R. A., Azenha M. A. (2024). Recent Advances in Light-Driven Semiconductor-Based
Micro/Nanomotors: Optimization Strategies and Emerging Applications. Molecules.

[90] Du S., Wang H., Zhou C., Wang W., Zhang Z. (2020). Motor and
Rotor in One: Light-Active ZnO/Au Twinned Rods of Tunable Motion Modes. J. Am. Chem. Soc..

[91] Chen X., Zhou C., Peng Y., Wang Q., Wang W. (2020). Temporal Light
Modulation of Photochemically Active, Oscillating Micromotors: Dark
Pulses, Mode Switching, and Controlled Clustering. ACS Appl. Mater. Interfaces.

[92] Liang Z., Teal D., Fan D. (2019). Light Programmable
Micro/Nanomotors
with Optically Tunable in-Phase Electric Polarization. Nat. Commun..

[93] Li J., Dekanovsky L., Khezri B., Wu B., Zhou H., Sofer Z. (2022). Biohybrid Micro- and Nanorobots for Intelligent Drug Delivery. Cyborg Bionic Syst..

[94] Blakemore R. (1975). Magnetotactic
Bacteria. Sci. New Ser..

[95] Felfoul O., Mohammadi M., Taherkhani S., De Lanauze D., Zhong Xu Y., Loghin D., Essa S., Jancik S., Houle D., Lafleur M., Gaboury L., Tabrizian M., Kaou N., Atkin M., Vuong T., Batist G., Beauchemin N., Radzioch D., Martel S. (2016). Magneto-Aerotactic
Bacteria Deliver Drug-Containing Nanoliposomes to Tumour Hypoxic Regions. Nat. Nanotechnol..

[96] Loghin D., Tremblay C., Mohammadi M., Martel S. (2017). Exploiting the Responses
of Magnetotactic Bacteria Robotic Agents to Enhance Displacement Control
and Swarm Formation for Drug Delivery Platforms. Int. J. Robot. Res..

[97] Fang R. H., Hu C.-M. J., Luk B. T., Gao W., Copp J. A., Tai Y., O’Connor D. E., Zhang L. (2014). Cancer Cell Membrane-Coated
Nanoparticles for Anticancer Vaccination and Drug Delivery. Nano Lett..

[98] Savchenko I. V., Zlotnikov I. D., Kudryashova E. V. (2023). Biomimetic Systems Involving Macrophages
and Their Potential for Targeted Drug Delivery. Biomimetics.

[99] Magdanz V., Khalil I. S. M., Simmchen J., Furtado G. P., Mohanty S., Gebauer J., Xu H., Klingner A., Aziz A., Medina-Sánchez M., Schmidt O. G., Misra S. (2020). IRONSperm:
Sperm-Templated Soft Magnetic Microrobots. Sci.
Adv..

[100] Xu H., Medina-Sánchez M., Magdanz V., Schwarz L., Hebenstreit F., Schmidt O. G. (2018). Sperm-Hybrid Micromotor for Targeted
Drug Delivery. ACS Nano.

[101] Wang X., Ma S., Liu R., Zhang T., Mao X., Chen Y., Wan P., Chi Z., Kong F. (2025). Microalgae-Driven
Microrobots: Revolutionizing Drug Delivery and Targeted Therapy in
Biopharmaceuticals. Adv. Biotechnol..

[102] Zhang F., Li Z., Duan Y., Luan H., Yin L., Guo Z., Chen C., Xu M., Gao W., Fang R. H., Zhang L., Wang J. (2022). Extremophile-Based
Biohybrid Micromotors for Biomedical Operations in Harsh Acidic Environments. Sci. Adv..

[103] Krishnababu K., Kulkarni G. S., Shetty Y. R. A., Rakesh
Babu S. N. (2023). Development of Micro/Nanobots and Their Application
in Pharmaceutical and Healthcare Industry. J.
Community Pharm. Pract..

[104] Zarepour A., Khosravi A., Iravani S., Zarrabi A. (2024). Biohybrid
Micro/Nanorobots: Pioneering the Next Generation of Medical Technology. Adv. Healthc. Mater..

[105] Zhang F., Guo Z., Li Z., Luan H., Yu Y., Zhu A. T., Ding S., Gao W., Fang R. H., Zhang L., Wang J. (2024). Biohybrid Microrobots
Locally and
Actively Deliver Drug-Loaded Nanoparticles to Inhibit the Progression
of Lung Metastasis. Sci. Adv..

[106] Zarepour A., Khosravi A., Iravani S., Zarrabi A. (2024). Biohybrid
Micro/Nanorobots: Pioneering the Next Generation of Medical Technology. Adv. Healthc. Mater..

[107] Wang, Y. D. Applied Sciences and Engineering: Modern Topics in Manufacturing and Designing; Trans Tech Publications Ltd., 2012; Vol. 197, 10.4028/b-c9lMoF.

[108] Zhang F., Li Z., Chen C., Luan H., Fang R. H., Zhang L., Wang J. (2024). Biohybrid
Microalgae
Robots: Design, Fabrication, Materials, and Applications. Adv. Mater..

[109] Sun T., Chen J., Zhang J., Zhao Z., Zhao Y., Sun J., Chang H. (2024). Application of Micro/Nanorobot in Medicine. Front. Bioeng. Biotechnol..

[110] Patra D., Sengupta S., Duan W., Zhang H., Pavlick R., Sen A. (2013). Intelligent, Self-Powered,
Drug Delivery
Systems. Nanoscale.

[111] Liu C., Hu Y., Lin J., Fu H., Lim L. Y., Yuan Z. (2019). Targeting Strategies for Drug Delivery
to the Kidney: From Renal
Glomeruli to Tubules. Med. Res. Rev..

[112] Kavousinejad, S. Simulation of Nanorobots with Artificial Intelligence and Reinforcement Learning for Advanced Cancer Cell Detection and Tracking. arXiv Preprint, arXiv:2411.02345, 2024.

[113] Sharma P. K., Chen C.-Y. (2025). AI-Integrated Micro/Nanorobots for
Biomedical Applications: Recent Advances in Design, Fabrication, and
Functions. Biosensors.

[114] Von Rohr, A. ; Trimpe, S. ; Marco, A. ; Fischer, P. ; Palagi, S. Gait Learning for Soft Microrobots Controlled by Light Fields. In 2018 IEEE/RSJ. International Conference on Intelligent Robots and Systems (IROS); IEEE: Madrid, 2018; pp 6199–6206. 10.1109/IROS.2018.8594092.

[115] Medany M., Piglia L., Achenbach L., Mukkavilli S. K., Ahmed D. (2025). Model-Based Reinforcement Learning
for Ultrasound-Driven Autonomous Microrobots. Nature Machine Intelligence.

[116] Tiryaki M. E., Demir S. O., Sitti M. (2022). Deep Learning-Based
3D Magnetic Microrobot Tracking Using 2D MR Images. IEEE Robot. Autom. Lett..

[117] Almaghthawi A., He C., Luo S., Alam F., Roshanfar M., Cheng L. (2026). Benchmarking Robust
AI for Microrobot
Detection with Ultrasound Imaging. Actuators.

[118] Botros K., Alkhatib M., Folio D., Ferreira A. (2023). USMicroMagSet:
Using Deep Learning Analysis to Benchmark the Performance of Microrobots
in Ultrasound Images. IEEE Robot. Autom. Lett..

[119] Dillinger C., Rasaiah A., Vogel A., Bahou C., Monastyrskaya K., Gheinani A. H., Ahmed D. (2025). Real-Time Color Flow
Mapping of Ultrasound Microrobots. Sci. Adv..

[120] Wrede P., Degtyaruk O., Kalva S. K., Deán-Ben X. L., Bozuyuk U., Aghakhani A., Akolpoglu B., Sitti M., Razansky D. (2022). Real-Time 3D Optoacoustic
Tracking
of Cell-Sized Magnetic Microrobots Circulating in the Mouse Brain
Vasculature. Sci. Adv..

[121] Zhang Y., Zheng M., Liu L., Dong G., Xiao R., Huang T. (2026). Spray-Based μ-LPBF
with Machine
Learning-Guided Track Morphology Prediction and Optimization. Int. J. Adv. Manuf. Technol..

[122] Salehi A., Hosseinpour S., Tabatabaei N., Soltani Firouz M., Zadebana N., Nauber R., Medina-Sánchez M. (2025). Advancements
in Machine Learning for Microrobotics in Biomedicine. Adv. Intell. Syst..

[123] Jiang J., Yang L., Zhang L. (2025). An Overview
of Micro/Nanorobot
Swarm Control: From Fundamental Understanding to Autonomy. IEEEASME Trans. Mechatron..

[124] Li M., Li L., Zhou J., Liu L., Jiao N. (2025). Deep Learning-Based
Automatic Control of Magnetic Diatom Biohybrid Microrobots for Targeted
Delivery. IEEE Trans. Robot..

[125] Glatt M., Sinnwell C., Yi L., Donohoe S., Ravani B., Aurich J. C. (2021). Modeling and Implementation
of a
Digital Twin of Material Flows Based on Physics Simulation. J. Manuf. Syst..

[126] Salehi A., Hosseinpour S., Tabatabaei N., Soltani Firouz M., Yu T. (2024). Intelligent Navigation of a Magnetic
Microrobot with Model-Free Deep Reinforcement Learning in a Real-World
Environment. Micromachines.

[127] Yang L., Jiang J., Gao X., Wang Q., Dou Q., Zhang L. (2022). Autonomous Environment-Adaptive
Microrobot Swarm Navigation
Enabled by Deep Learning-Based Real-Time Distribution Planning. Nat. Mach. Intell..

[128] Bharadwaj, S. ; Almekkawy, M. Deep Learning Based Motion Tracking of Ultrasound Image Sequences. In 2020 IEEE International Ultrasonics Symposium (IUS); IEEE: Las Vegas, NV, USA, 2020; pp 1–4. 10.1109/IUS46767.2020.9251739.

[129] Yang L., Jiang J., Ji F., Li Y., Yung K.-L., Ferreira A., Zhang L. (2024). Machine Learning for
Micro- and Nanorobots. Nat. Mach. Intell..

[130] Dong H., Lin J., Tao Y., Jia Y., Sun L., Li W. J., Sun H. (2024). AI-Enhanced Biomedical Micro/Nanorobots
in Microfluidics. Lab. Chip.

