# Supplementary material for: Micro/Nanorobots (MNRs) at the Intersection of Mechanobiology and Artificial Intelligence: An Emerging Frontier for Precision Nanomedicine
Source: ACS Nano Med. 2026 Jun 9;1(8):1706–24. doi: 10.1021/acsnanomed.5c00168 (PMC13564963; doi:10.1021/acsnanomed.5c00168)
Supplement: Supplementary file 1 [file nm5c00168_si_001.pdf]

SUPPORTING INORMATION

Micro/Nano Robots (MNRs) at the Intersection of Mechanobiology and Artificial Intelligence: An Emerging Frontier for Precision Nanomedicine

Tahniat Afsari[†,#], Jamile Harmouch[†], Rahul Sharma[§], Ruisheng Liu[‡],
Subhra Mohapatra[#,‖,*] and Shyam S. Mohapatra[†,‖,*]

† Department of Internal Medicine and Laboratory for Translational Nanotechnology, Morsani College of Medicine, Tampa, FL 33612, USA
# Department of Molecular Medicine, Morsani College of Medicine, Tampa, FL 33612, USA
§ Division of Nephrology, Center for Immunity Inflammation and Regenerative Medicine University of Virginia School of Medicine, Charlottesville, VA, 22903 USA
‡ Department of Molecular Pharmacology and Physiology, Morsani College of Medicine, Tampa, FL 33612, USA
‖ James A. Haley VA Hospital, Tampa, FL 33612, USA

[*]*Corresponding authors:* Shyam S. Mohapatra (smohapat@usf.edu) and Subhra Mohapatra (smohapa2@usf.edu)

Table S1. A Summary of Various Propulsion Modes and Their Corresponding Energy Sources for Actuation, Control a or Modeling Approaches Key Advantages for Nanomedicine and Primary Limitations and Translational Challenges.

**Propulsion Mode**	**Energy Source / Actuation**	**Representative Control or Modeling Approaches**	**Dominant Mechanical Signature at Nano–Bio Interface**	**Key Advantages for Nanomedicine**	**Primary Limitations / Translational Challenges**
Magnetic Actuation	External magnetic fields	Torque–drag models; kinematic and predictive control[1–4]	Rotational shear and surface stress[34]	High control precision; deep tissue penetration[13]	External hardware; long-term biocompatibility[29]
Acoustic / Ultrasound Actuation	Ultrasound waves	Acoustic field and FSI models[56]	Oscillatory shear and microstreaming[5]	Noninvasive; barrier modulation[56]	Attenuation; cavitation risk[56]
Catalytic (Chemical) Propulsion	Local chemical reactions	Reaction–diffusion and stochastic models[78]	Reaction-driven thrust and shear[7]	Autonomous propulsion[78]	Limited control; fuel dependence[78]
Optical / Photothermal Actuation	Visible/NIR light	Thermophoretic and optical modeling[26]	Localized thermal stress[2]	High spatial precision[26]	Limited penetration; thermal damage[2]
Biohybrid Propulsion	Cellular motility	Hybrid biological–physical control[10]	Low-shear cell-mediated forces[10]	High biocompatibility[10]	Biological variability[10]
Swarm-Based Collective Propulsion	Collective actuation	Multi-agent learning and control[11]	Collective shear amplification[11]	Enhanced robustness and force[11]	Emergent safety risks[11]

References

1. Nelson, B. J.; Kaliakatsos, I. K.; Abbott, J. J. *Microrobots for Minimally Invasive Medicine.* Annu. Rev. Biomed. Eng. 2010, 12, 55–85. https://doi.org/10.1146/annurev-bioeng-010510-103409
2. Li, J.; Esteban-Fernández de Ávila, B.; Gao, W.; Zhang, L.; Wang, J. *Micro/Nanorobots for Biomedicine: Delivery, Surgery, Sensing, and Detoxification.* Sci. Robot. 2017, 2, eaam6431. https://doi.org/10.1126/scirobotics.aam6431

3. Tottori, S.; Zhang, L.; Qiu, F.; Krawczyk, K. K.; Franco-Obregón, A.; Nelson, B. J. *Magnetic Helical Micromachines: Fabrication, Controlled Swimming, and Cargo Transport.* Adv. Mater. 2012, 24, 811–816. https://doi.org/10.1002/adma.201103818
4. Servant, A.; Qiu, F.; Mazza, M.; Kostarelos, K.; Nelson, B. J. *Controlled In-Vivo Swimming of a Swarm of Bacteria-Like Microrobotic Flagella.* Adv. Mater. 2015, 27, 2981–2988. https://doi.org/10.1002/adma.201500225
5. Ahmed, D.; Lu, M.; Nourhani, A.; Lammert, P. E.; Stratton, Z.; Muddana, H. S.; Crespi, V. H.; Huang, T. J. *Selective Manipulation of Particles by Acoustic Microstreaming.* Lab Chip 2015, 15, 204–212. https://doi.org/10.1039/C4LC01215J
6. Wu, Z.; Chen, Y.; Mukasa, D.; Pak, O. S.; Gao, W. *Medical Micro/Nanorobots in Complex Media.* Chem. Soc. Rev. 2020, 49, 8088–8112. https://doi.org/10.1039/D0CS00309C
7. Wang, H.; Pumera, M. *Fabrication of Micro/Nanoscale Motors.* Chem. Rev. 2015, 115, 8704–8735. https://doi.org/10.1021/cr500572x
8. Sánchez, S.; Soler, L.; Katuri, J. *Chemically Powered Micro- and Nanomotors.* Angew. Chem., Int. Ed. 2015, 54, 1414–1444. https://doi.org/10.1002/anie.201406096
9. Zhou, H.; Mayorga-Martinez, C. C.; Pané, S.; Zhang, L.; Pumera, M. *Magnetic Micro- and Nanorobots for Environmental and Biomedical Applications.* Chem. Rev. 2021, 121, 4999–5041. https://doi.org/10.1021/acs.chemrev.0c00987
10. Park, B.-W.; Zhuang, J.; Yasa, O.; Sitti, M. *Multifunctional Bacteria-Driven Microswimmers for Targeted Active Drug Delivery.* ACS Nano 2017, 11, 8910–8923. https://doi.org/10.1021/acsnano.7b04707
11. Li, X.; Xu, T.; Wang, X.; Zhang, L. *Swarm Intelligence in Medical Microrobots.* Adv. Intell. Syst. 2022, 4, 2100257. https://doi.org/10.1002/aisy.202100257

Integration of multichannel immunofluorescent (IF) imaging with spatial, molecular, and experimental metadata.

Especially for MNR–induced mechanotransduction studies, hybrid multimodal CNN–GNN (graph neural network) frameworks provide a powerful paradigm for integrating multichannel immunofluorescent (IF) imaging with spatial, molecular, and experimental metadata.[112] In this approach, CNNs are first employed to extract rich, hierarchical features from IF images, where multiple fluorescence channels capture mechanosensitive markers such as integrins, focal adhesion proteins, cytoskeletal components, and nuclear mechanotransducers (e.g., YAP/TAZ, ERK). CNNs excel at quantifying subcellular morphology, protein localization, and intensity patterns directly from pixel level data, enabling robust characterization of local cellular responses to MNR applied forces. These learned image features are then integrated into GNNs, where individual cells are represented as nodes enriched with CNN derived embeddings

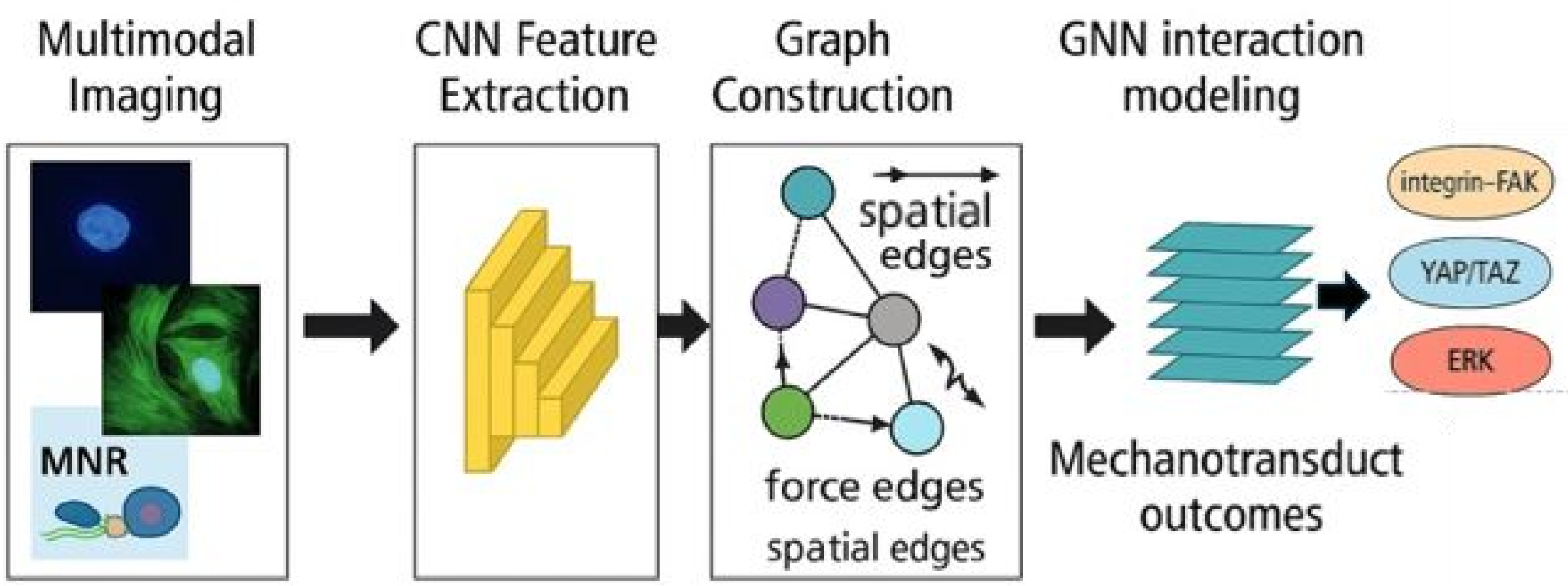

Figure S1. A schematic illustrating a hybrid CNN–GNN architecture for modeling magnetic and acoustic micro/nanorobot–induced mechanotransduction. Multimodal imaging and mechanical signals are encoded using convolutional neural networks, followed by force aware graph construction and temporal modeling with recurrent or transformer modules. Graph neural networks integrate dynamic spatial interactions to predict activation of integrin–FAK, YAP/TAZ, and ERK signaling pathways.

and molecular measurements, while edges encode spatial proximity, cell–cell contacts, or MNR-mediated force coupling. This graph-based representation allows explicit modeling of how mechanical cues propagate across cellular neighborhoods, capturing collective and emergent behaviors that are central to mechanotransduction but difficult to resolve with image only models. By fusing IF image features with spatial relationships and contextual metadata (such as actuation parameters, time points, or cell type), multimodal CNN–GNN architectures enable interpretable, pathway level inference of mechanotransducive signaling, linking MNR induced physical perturbations to coordinated activation of integrin–FAK, YAP/TAZ, and ERK pathways at the tissue or microenvironment scale.
